# Opportunistic Pathogens in Drinking Water Distribution Systems—A Review

**DOI:** 10.3390/microorganisms12050916

**Published:** 2024-04-30

**Authors:** Mark W. LeChevallier, Toby Prosser, Melita Stevens

**Affiliations:** 1 Dr. Water Consulting, LLC., Morrison, CO 80465, USA; 2 Melbourne Water, Melbourne, VIC 3001, Australia; toby.prosser@melbournewater.com.au (T.P.); melita.stevens@melbournewater.com.au (M.S.)

**Keywords:** opportunistic pathogens, drinking water, distribution systems, legionella pneumophila, mycobacterium, pseudomonas, climate change, disinfection, flushing, water treatment

## Abstract

In contrast to “frank” pathogens, like *Salmonella entrocolitica*, *Shigella dysenteriae*, and *Vibrio cholerae*, that always have a probability of disease, “opportunistic” pathogens are organisms that cause an infectious disease in a host with a weakened immune system and rarely in a healthy host. Historically, drinking water treatment has focused on control of frank pathogens, particularly those from human or animal sources (like *Giardia lamblia*, *Cryptosporidium parvum*, or *Hepatitis A virus*), but in recent years outbreaks from drinking water have increasingly been due to opportunistic pathogens. Characteristics of opportunistic pathogens that make them problematic for water treatment include: (1) they are normally present in aquatic environments, (2) they grow in biofilms that protect the bacteria from disinfectants, and (3) under appropriate conditions in drinking water systems (e.g., warm water, stagnation, low disinfectant levels, etc.), these bacteria can amplify to levels that can pose a public health risk. The three most common opportunistic pathogens in drinking water systems are *Legionella pneumophila*, *Mycobacterium avium*, and *Pseudomonas aeruginosa*. This report focuses on these organisms to provide information on their public health risk, occurrence in drinking water systems, susceptibility to various disinfectants, and other operational practices (like flushing and cleaning of pipes and storage tanks). In addition, information is provided on a group of nine other opportunistic pathogens that are less commonly found in drinking water systems, including *Aeromonas hydrophila*, *Klebsiella pneumoniae*, *Serratia marcescens*, *Burkholderia pseudomallei*, *Acinetobacter baumannii*, *Stenotrophomonas maltophilia*, *Arcobacter butzleri*, and several free-living amoebae including *Naegleria fowleri* and species of *Acanthamoeba.* The public health risk for these microbes in drinking water is still unclear, but in most cases, efforts to manage *Legionella*, mycobacteria, and *Pseudomonas* risks will also be effective for these other opportunistic pathogens. The approach to managing opportunistic pathogens in drinking water supplies focuses on controlling the growth of these organisms. Many of these microbes are normal inhabitants in biofilms in water, so the attention is less on eliminating these organisms from entering the system and more on managing their occurrence and concentrations in the pipe network. With anticipated warming trends associated with climate change, the factors that drive the growth of opportunistic pathogens in drinking water systems will likely increase. It is important, therefore, to evaluate treatment barriers and management activities for control of opportunistic pathogen risks. Controls for primary treatment, particularly for turbidity management and disinfection, should be reviewed to ensure adequacy for opportunistic pathogen control. However, the major focus for the utility’s opportunistic pathogen risk reduction plan is the management of biological activity and biofilms in the distribution system. Factors that influence the growth of microbes (primarily in biofilms) in the distribution system include, temperature, disinfectant type and concentration, nutrient levels (measured as AOC or BDOC), stagnation, flushing of pipes and cleaning of storage tank sediments, and corrosion control. Pressure management and distribution system integrity are also important to the microbial quality of water but are related more to the intrusion of contaminants into the distribution system rather than directly related to microbial growth. Summarizing the identified risk from drinking water, the availability and quality of disinfection data for treatment, and guidelines or standards for control showed that adequate information is best available for management of *L. pneumophila*. For *L. pneumophila*, the risk for this organism has been clearly established from drinking water, cases have increased worldwide, and it is one of the most identified causes of drinking water outbreaks. Water management best practices (e.g., maintenance of a disinfectant residual throughout the distribution system, flushing and cleaning of sediments in pipelines and storage tanks, among others) have been shown to be effective for control of *L. pneumophila* in water supplies. In addition, there are well documented management guidelines available for the control of the organism in drinking water distribution systems. By comparison, management of risks for *Mycobacteria* from water are less clear than for *L. pneumophila*. Treatment of *M. avium* is difficult due to its resistance to disinfection, the tendency to form clumps, and attachment to surfaces in biofilms. Additionally, there are no guidelines for management of *M. avium* in drinking water, and one risk assessment study suggested a low risk of infection. The role of tap water in the transmission of the other opportunistic pathogens is less clear and, in many cases, actions to manage *L. pneumophila* (e.g., maintenance of a disinfectant residual, flushing, cleaning of storage tanks, etc.) will also be beneficial in helping to manage these organisms as well.

## 1. Introduction

Fundamentally, there are three basic mechanisms by which pathogenic microorganisms occur in treated drinking water: (1) microbes break through (or are not treated by) the treatment process from the source water supply, (2) microbes regrow, typically in biofilms, from very low initial levels, and (3) organisms result from a recontamination of the treated water (via intrusion or cross connections) within the distribution pipeline system. These mechanisms are incorporated in the concept of multiple barriers for water treatment, the cornerstone of sanitary engineering. These barriers are selected to duplicate removal capabilities by succeeding process steps. In this way, sufficient backup systems are available to permit continuous operation in the face of normal mechanical failures. Traditionally, the barriers have included:

- Source water protection
- Coagulation, flocculation, sedimentation, and filtration
- Disinfection
- Protection of the distribution system

Where one of the barriers is missing (e.g., no source water protection or lack of filtration), it is important to strengthen the other barriers in the process.

Historically, water treatment for microbial agents has primarily focused on treatment and “disinfection” of frank (organisms that always cause disease) enteric pathogens in the source water [1]. Tap water has never been considered sterile and disinfection of source water frank pathogens has the objective to reduce pathogenic microbes to “acceptable” or “tolerable” levels of risk as defined by national or international regulations. Disinfection can be achieved through a variety of chemical or physical means (i.e., chlorination or ultraviolet light) and may require the removal of particulate or organic material depending on the source water quality. A secondary objective of water treatment is to deliver the treated and disinfected water safely to the customer with minimal degradation of water quality or recontamination by pathogenic organisms.

Definitions:

- “frank” pathogens are microbes that always cause disease.
- “opportunistic” pathogens are organism that cause an infectious disease in a host with a weakened immune system.

**Figure 1:**
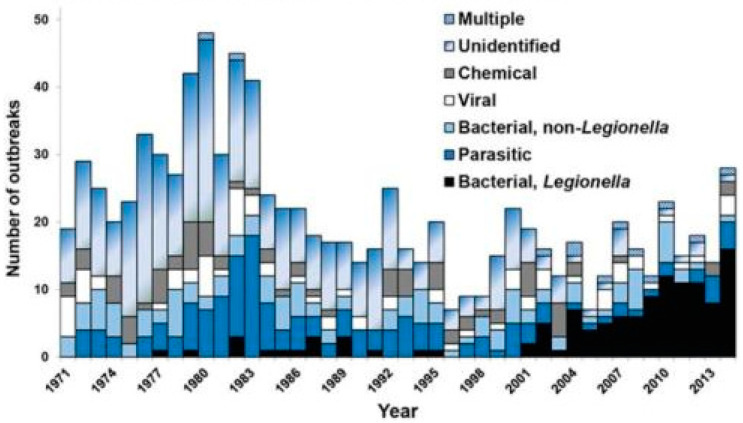
Etiology of drinking water associated outbreaks (*n* = 928) by year, US, 1971–2014. Source: Benedict et al. [9].

At the beginning of the last century (1900s), the focus of water treatment was on controlling bacterial pathogens like typhoid and cholera [2]. In the 1950s and 1960s, the focus was on controlling viral pathogens like poliovirus and hepatitis A virus [3]. In the 1980s and 1990s, the attention turned towards protozoan pathogens like *Giardia* and *Cryptosporidium* [4]. However, in the past 20 years, outbreaks from these fecal-oral pathogens have been rare but outbreaks due to *Legionella pneumophila* and other opportunistic pathogens have increased (an opportunistic pathogen is an organism that causes an infectious disease in a host with a weakened immune system). For example, in the United States (USA), outbreaks due to *Legionella* species (spp.) have increased nearly 10-fold over the past 20 years (Figure 1). This increase is not just associated with *Legionella* spp. Donohue [5,6] reviewed clinical laboratory reports and found that clinical cases of mycobacteria doubled from 8.2 per 100,000 persons in 1994 to 16 per 100,000 persons in 2014. Collier et al. [7] estimated the public health burden for waterborne diseases (from all sources—not just drinking water) and showed that the highest overall healthcare costs were associated with illnesses caused by nontuberculous mycobacteria, *Pseudomonas*, and *Legionella*. This trend is not just associated with the USA; similar reports show increases in waterborne disease in Europe and Australia [8].

The challenge for controlling opportunistic waterborne pathogens is not just providing adequate treatment of the source water but managing the many kilometers of the water network all the way to the consumer’s tap. This sets up a “shared responsibility” between the water utility and the building owner, as the management of the building water plumbing is typically outside of the responsibility of the water utility. Management activities to limit opportunistic pathogens in utility-operated distribution systems have not been explicitly regulated by national authorities, although several initiatives are under development [10,11,12]. Importantly, there are fundamental differences between fecal-oral pathogens and the opportunistic pathogens like *L. pneumophila*, *Mycobacterium avium*, and *Pseudomonas aeruginosa*:

1. Opportunistic pathogens are normal inhabitants of the aquatic environment. It does not require an event, like fecal contamination, for the bacteria to be present.
2. Unlike most fecal-oral pathogens, opportunistic pathogens can grow in water provided the right temperature and conditions are present.
3. The ecology of many of the opportunistic pathogens are more complex than fecal pathogens and often involves intracellular growth in free living amoebae that can amplify the bacterial virulence factors, leading to human infections.
4. Drinking water can be an important route of exposure for opportunistic pathogens, but often other uses of water are also important, including cooling towers, hot tubs and pools, ornamental fountains, and industrial equipment [13].
5. The routes of infection for opportunistic pathogens in drinking water often go beyond simple ingestion and include inhalation, dermal exposure, ocular, and acute otitis externa (outer ear infection).
6. The infectious dose for opportunistic pathogens is often much higher than fecal-oral pathogens, meaning that the concentration of these organisms in water is much more important than just their mere presence.
7. Generally, opportunistic pathogens pose the greatest risk to the elderly, the immunocompromised (e.g., those undergoing cancer treatment, organ transplant, or in intensive care units), or those with some underlying risk factor (e.g., smoking, alcohol consumption, pregnancy, asthma, etc.). However, in some cases the very young can also be at risk.

It should be noted that drinking water is not the only route of infection for most opportunistic pathogens and national and international guidelines [1,14] recommend that that people with underlying health issues should seek medical advice about protecting themselves from opportunistic pathogens.

The three most common opportunistic pathogens in drinking water systems are *L. pneumophila*, *M. avium*, and *P. aeruginosa*. This report focuses on these organisms to provide information on their public health risk, occurrence in drinking water systems, susceptibility to various disinfectants, and other operational practices (like flushing and cleaning of pipes and storage tanks). In addition, information is provided on a group of nine other opportunistic pathogens that are less commonly found in drinking water systems, including *Aeromonas hydrophila*, *Klebsiella pneumoniae*, *Serratia marcescens*, *Burkholderia pseudomallei*, *Acinetobacter baumannii*, *Stenotrophomonas maltophilia*, *Arcobacter butzleri*, and several free-living amoebae including *Naegleria fowleri* and species of *Acanthamoeba.* The public health risk for these microbes in drinking water is still unclear, but in most cases, efforts to manage *Legionella*, mycobacteria, and *Pseudomonas* risks will also be effective for these other opportunistic pathogens.

The objective of this report is to explore the factors that help control the occurrence and concentrations of opportunistic pathogens in drinking water distribution systems. It is important to note that most outbreaks of opportunistic pathogens typically occur in building plumbing—and not the portion of the distribution system under the control of utilities—but it is critical for utilities to manage their systems so that water of the highest quality can be delivered to their customers. Many of the principles outlined in this report can also be used in a building water management program, but there are also other considerations in building plumbing that are outside the scope of this study (excellent guidance is available for building systems; see [15,16,17,18]). The fundamental theme that will run throughout this review focuses on the control of the growth of opportunistic pathogens through the implementation of an effective distribution system management program.

## 2. Impact of Climate Change on the Microbiology of the Distribution System

Climate change has a fundamental impact on the microbiology of drinking water distribution systems, affecting both the physicochemical and operational aspects of treatment. From too little water to too much water; from severe weather to changes in water quality; climate change impacts all the critical areas that are important to providing safe, clean, and efficient water services. Damage to utility infrastructure can result in prolonged operational impacts and environmental contamination. For example, nutrients and contaminants of emerging concern (CECs) were detected in shallow groundwater downgradient of New Jersey and New York coastal onsite wastewater disposal systems following Hurricane Sandy [19]. As with Hurricane Sandy and other severe weather events, water utility operations, including electrical power, chemical delivery, communications etc., can be disrupted. Over 2.3 million Puerto Rican residents were served by water systems contaminated by bacteria after Hurricane Maria struck the island in 2018 and many small water systems were severely damaged and inoperable even months after the disaster [20]. In coastal areas, increases in sea-level and decreases in groundwater recharge due to climate change can leave billions of people worldwide without a reliable source of water [21].

The danger of fires in the watershed is another increased risk posed by climate change. Ash and residue from wildfires can increase the organic content and suspended solids of the source waters [22]. The 2019–2020 wildfire season in Australia was unprecedented in recorded history, burning several catchments supplying drinking water to the 5.5 million inhabitants of Sydney [23]. Modelling has indicated that a large wildfire in the Upper Yarra Reservoir, followed by a storm, could result in water being untreatable for a year or more [24]. Depending on the intensity of the fire, levels of organic carbon, phosphorus, nitrogen, heavy metals (e.g., chromium, arsenic, lead, mercury, and copper), cyanide, polycyclic aromatic hydrocarbons (PAH), polychlorinated biphenyls (PCB), polychlorinated dibenzopdioxins and dibenzofurans (PCDD/F), and various minerals (sodium, magnesium, calcium, potassium, chloride, and sulfate) can be increased in the water supply.

Increased risk from waterborne outbreaks (from enteric/frank pathogens) and changes in water quality can be impacted by climate change which can drive extremes in precipitation that result both in heavy rainfall events and periods of drought. Curriero et al. [25] reported that extreme rainfall events were associated with increased risk of waterborne outbreaks and showed that 51% of waterborne disease outbreaks were preceded by precipitation events above the 90th percentile (the 10% most intense events), and 68% by events above the 80th percentile. Outbreaks due to surface water contamination showed the strongest association with extreme precipitation during the month of the outbreak, whereas groundwater contamination showed an average two-month lag. The leading edge of a hydrograph (termed “first flush”) can contain high concentrations of contaminants that can overwhelm treatment barriers. Atherholt et al. [26] reported *Cryptosporidium* levels were more than 100 times higher in the first flush following heavy rainfall than prior to the event. Droughts, too, can accumulate contaminants on impervious surfaces that are released during subsequent rainfalls. Nichols et al. [27] reported that 89 outbreaks of *Giardia*, *Cryptosporidium*, *E. coli*, *S. typhi*, *S. paratyphi*, *Campylobacter*, and *Streptobacillus moniliformis* in England and Wales were preceded by periods of either heavy rainfall or low rainfall. Low stream flows during dry periods can increase the proportion of wastewater in the receiving waters—increasing the contaminant loads, levels of nitrogen and phosphorus, and pathogens [28]. Increased temperatures associated with climate change, along with nitrogen and phosphorus in the runoff, can trigger algal blooms and the release of algal toxins that can cause water treatment challenges and pose animal and human health risks. Algal blooms can increase levels of easily assimilated organic carbon (AOC), a nutrient linked to accelerated biofilm growth in distribution systems [29]. Increased temperatures can stimulate biological activity within the distribution system and accelerate chemical reactions including disinfectant decay and increased corrosion. Increased wet weather and air humidity has been associated with increased *L. pneumophila* transmission and risk [30].

Climate change can be associated with higher water temperatures, which can affect all the processes involved in microbiological water quality: microbial growth rate, disinfection efficiency, decay of disinfectant residual, corrosion rates, and distribution system hydraulics (increased water velocity from increased consumer demand) [31]. On average, bacterial growth in distributed water is significantly higher when water temperatures are greater than 15 °C [32,33]. *L. pneumophila* was detected in drinking water distribution systems only at temperatures greater than 18 °C [34]. In recent excellent reviews on the impact of climate change on opportunistic pathogens in water systems, O’Keeffe [35] and Blanc et al. [36] provide evidence that increases in water temperature will increase the proliferation of *Legionella* spp., *Mycobacterium* spp., and other opportunistic pathogens. A pilot study by Calero-Preciado et al. [37] demonstrated increases in opportunistic pathogens (*M. avium*, *P. aeruginosa*, *Acanthamoeba*, and *S. maltophilia*) in biofilm and water samples when water temperatures increased from 16 to 24 °C. O’Keeffe [35] notes that warmer temperatures would increase the use of cooling towers that could promote *Legionella* risk. Increased water conservation efforts due to climate change would accelerate the adoption of water efficient devices that could indirectly promote the growth of opportunistic pathogens through increased water age and decreased disinfectant residuals [35]. Disease risk for some *Mycobacterium* species can be greater in warmer or tropical regions [36].

Climate change can affect source water concentrations of opportunistic pathogens too. *N. fowleri* in surface water is associated with temperatures >25 °C and scientists associate the greater distribution of this microbe to increases in temperature due to climate change [38,39,40]. Kemble et al. [38] attributed an infection of *N. fowleri* from surface water in Minnesota, USA, to warm water temperatures due to climate change where water temperatures averaged 25 °C; 3.6 °C above normal. Algal blooms associated with more intense sunlight and warmer temperatures can serve as a source of nutrients for opportunistic pathogens. Cyanobacteria are extensively grazed by amoebae, and *Naegleria* (and other free-living amoeba) have been associated with cyanobacteria-dominated layers in a stratified lake [39]. Source water concentrations of *L. pneumophila*, *B. pseudomallei*, and other opportunistic pathogens can be expected to increase with warmer water temperatures associated with climate change [35,36,41].

In summary, the trends in climate change—particularly the increases in extreme precipitation events and temperature–will increase the concerns related to opportunistic pathogens in water. Thus, it is prudent to evaluate how climate change will affect the risks of opportunistic pathogens in drinking water systems.

## 3. Primary Treatment Considerations

The types of processes used to treat source water can greatly impact the biological stability of and bacterial amplification in drinking water systems. Groundwaters are typically stable (e.g., little change in bacterial levels) due to the natural percolation of water through the soil environment that removes biodegradable organic matter. However, the presence of methane, ferrous iron, reduced sulfur compounds, hydrogen gas, manganese, ammonia, and nitrite can serve as either carbon or energy sources that can promote growth of certain microbes [42]. In some cases, excess ammonia levels have been related to serious bacterial growth problems [43,44].

Surface water treatment processes, including the type of coagulant, clarification process, filter media, and disinfection regime, can alter the biological stability of treated water [29,45]. Unfiltered surface water supplies were found to be particularly susceptible to bacterial growth [46]. Even though unfiltered supplies should have fully protected water sources, even low levels of particulate materials will accumulate in low flow portions of the distribution system producing sediments that can protect bacteria from disinfection. In water systems that supply thousands of megaliters per day, even micrograms of particles can result in kilograms of sediment if not routinely flushed out of the system. Organic particles and algae can decompose over an extended period, producing biodegradable material to support microbial growth [47]. Therefore, for all systems, but especially for unfiltered systems, a routine and aggressive unidirectional flushing program for distribution system mains and periodic inspection and cleaning of storage facilities is critical to minimizing bacterial growth in the system. In regions where water is scarce, the cost benefit of such flushing in a water scarce environment must be carefully weighed against other solutions such as filtration or technologies that can flush, filter, disinfect, and return the water back to the distribution system (see: https://www.no-des.com/ (accessed on 24 April 2024)).

The integrity of the disinfection barrier is paramount to providing safe drinking water. Increases in natural organic matter (NOM) or turbidity can create conditions that can impair primary disinfection [48]. Mycobacteria have hydrophobic mycolic acids in their cell walls [49], which favors their attachment to turbidity particles and other surfaces. Falkinham et al. [50] reported that the concentration of *M. avium* in raw surface water samples was significantly associated with turbidity levels > 2 NTU (r^2^ = 0.93, *p* < 0.0001) and that the detection rate of raw water *M. avium* was associated with turbidity levels > 2 NTU (r^2^ = 0.63, *p* < 0.02). Similarly, *L. pneumophila* can be encased in amoeba cysts and be highly resistant to disinfection [51]. *L. pneumophila* in protozoa cysts survived 25-fold more chlorine disinfection than planktonic cells after 18 h [52]. These results illustrate why catchment management is important. Appropriate land use management, placement and use of riparian buffers and flood plain management practices, wetlands, off-stream reservoirs, and reservoirs in series can help mitigate spikes in turbidity and degradation of water quality [53]. As mentioned previously, climate change can lead to extreme precipitation events that can cause high turbidity levels in “first flush” runoff that can impair disinfection barriers [26]. Extreme weather events can also cause momentary interruption of power that can lead to lapses in treatment. Because the disinfection barrier is the primary tool to remove pathogenic microbes from the water supply and protect public health, it is important that redundancy is provided to ensure its continuity of operation.

## 4. Factors Influencing Growth of Microbes in Distribution Systems

Since the management of opportunistic pathogens in water supplies is focused on the management of the growth of these organisms in biofilms in the distribution system, this section discusses the factors that influence the growth of microorganisms in drinking water. The coliform group has traditionally been used as an indicator of the integrity of treatment and the distribution system. While most of these indictor bacteria have low public health significance, amplification of these microbes could be used as an indicator that conditions are favorable for the growth of opportunistic pathogens in the distribution system. Therefore, efforts to manage the growth of coliform bacteria would be useful for opportunistic pathogen risk management.

**Figure 2:**
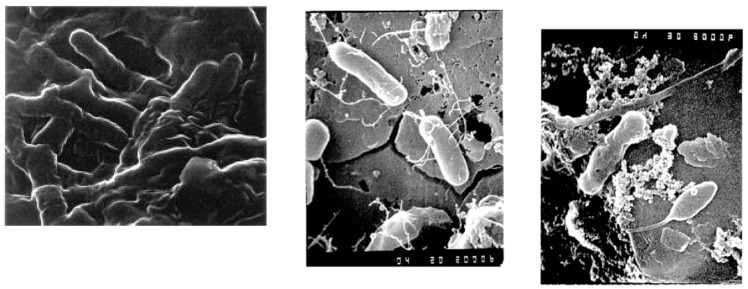
Examples of biofilms in water systems. From: LeChevallier [33].

Biofilms. Growth is the increase in bacterial numbers in the distribution system due to cell reproduction. Significant growth always occurs at the expense of an organic or inorganic substrate. Most microbial growth is thought to occur in biofilms on distribution pipe surfaces (Figure 2). Biofilm refers to an organic or inorganic deposit consisting of microorganisms, microbial products, and detritus at a surface [54]. Biofilms may occur on pipe surfaces, sediments, inorganic tubercles (a nodular or knobby outgrowth on a pipe surface), suspended particles, or virtually any substratum immersed in the aquatic environment. Biofilms provide an ecological advantage for microbial growth, including adhesion/cohesion capabilities, mechanical properties, nutritional sources, metabolite exchange platform, cellular communication, protection and resistance to biocides, environmental stresses (e.g., dehydration and ultraviolet light), host immune attacks (e.g., antibodies, complement system, antimicrobial peptides, and phagocytes), and shear forces [55]. Biofilms in drinking water pipe networks can be responsible for a wide range of water quality and operational problems. Biofilms contribute to loss of distribution system disinfectant residuals, increased bacterial levels, reduction of dissolved oxygen, taste, and odor changes, red or black water problems due to iron or sulfate-reducing bacteria, microbial-influenced corrosion, hydraulic roughness, and reduced materials life [33]. Management strategies for opportunistic pathogens in drinking water systems should consider both the control of these organisms in biofilms as well as the water column.

Temperature. We have previously discussed how climate change will accelerate issues with the impact of temperature on microbial growth in drinking water supplies. The Q_10_ temperature coefficient describes the factor by which the rate of a reaction (R) increases for every 10-degree rise in temperature (T). The Q_10_ is calculated as:

Q10=R2R1 10 ℃/(T2−T1)

Most biological systems have a Q_10_ between 2–3, and the coefficient can be site specific [56]. A Q_10_ of 2 means that the rate of the reaction doubles for each 10 °C rise in temperature; a Q_10_ of 3 means that the reaction rate triples with each 10 °C rise in temperature.

**Figure 3:**
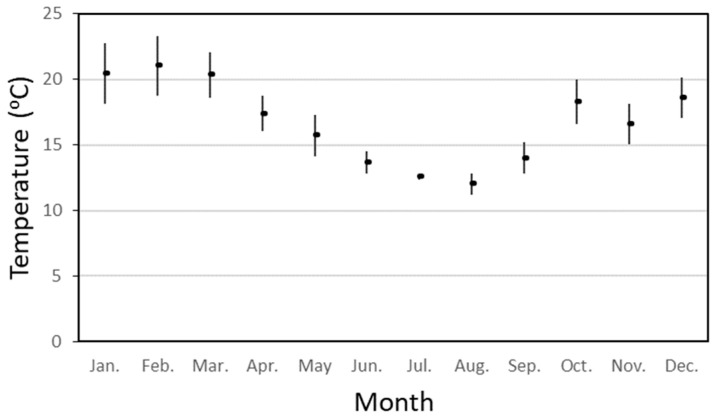
Average and standard deviation of water temperatures in treated water storages within a distribution system in Australia. Data from 2013–2022.

Figure 3 shows the average and standard deviation of water temperatures in treated water storages in a distribution system from Australia (based on data from 2013–2022). The highest temperatures are in the January to March period, where average temperatures exceed 20 °C and values can range up to 25 °C. The maximum water temperature during this period was 26.7 °C. The optimal temperature range for *L. pneumophila* to express factors that promote infection and transmission is between 25 °C and 30 °C (NASEM 2019). Likewise, *M. avium* grows at temperatures between 14 and 37 °C, with an optimum growth rate between 28 and 37 °C [57]. *P. aeruginosa* is a mesophilic bacterium, growing at temperatures ranging from 4 °C to over 42 °C, with an optimal growth temperature of 37 °C [58].

Besides being conducive for growth, elevated water temperatures also promote the decay of disinfectant residuals and accelerate corrosion of metallic surfaces. The decay of a chlorine residual can be described as a first order reaction that increases with temperature [59]. In one system studied, the chlorine decay coefficient increased over 50% when water temperatures increased less than 4 °C—from an average of 15.7 °C to 19.4 °C [59]. Of course, the decay of a free chlorine residual is also impacted by the concentration of organic carbon, certain inorganic chemicals (i.e., ammonia, iron, manganese, etc.), and pipe materials, among other factors, but this study illustrates the role of even minor temperature changes and the value of modeling chlorine decay within the distribution system [60,61].

Disinfectant Type and Concentration. Maintenance of a disinfectant residual is an integral part of a water management plan for control of opportunistic pathogens [15]. Disinfection methods should be paired and scheduled with water testing to ensure that the system is effective in managing disinfectant residuals to minimize microbial occurrence. Chemical disinfectants, particularly oxidizing agents such as chlorine, chlorine dioxide, or chloramine, are most widely used by utilities to manage microbial growth in distribution systems. The disinfectant should ideally be able to inactivate microorganisms in the bulk water but also penetrate and inactivate microorganisms associated with biofilms. Free chlorine and chlorine dioxide are strong disinfectants capable of rapid microbial inactivation, but chloramines are better at penetrating [31,62,63]. For example, LeChevallier et al. [46] reported that the occurrence of coliform bacteria growing in systems that used free chlorine was 0.97% of 33,196 samples, while 0.51% of 35,159 samples from chloraminated systems contained coliform bacteria (statistically different at *p* < 0.0001), and the average density of coliform bacteria was 35 times higher in free chlorinated systems as compared to chloraminated water (0.60 colony-forming units [cfu]/100 mL for free chlorinated water, compared to 0.017 cfu/100 mL for chloraminated water). The penetration of free chlorine into a biofilm has been shown to be limited by its fast reaction rate [64]. Essentially, free chlorine is consumed before it can react with the bacterial components of the film [65]. Chloramines, on the other hand, react more slowly and can diffuse into the biofilm and eventually inactivate attached bacteria. Stewart et al. [66] showed that free chlorine did not penetrate alginate beads containing bacterial cells, but chloramines did penetrate the alginate material and reduced bacterial levels nearly 1 million-fold over a 60-min interval (2.5 mg/L chloramines, pH 8.9).

The concentration of residual disinfectant is also important in maintaining microbial water quality in distribution systems. It is not just the average disinfectant residual that is important, but the concentration maintained at the end of the distribution system. For example, LeChevallier et al. [46] showed that systems that maintained dead-end free chlorine levels < 0.2 mg/L, or monochloramine levels < 0.5 mg/L, had substantially more growth of coliform bacteria than systems that maintained higher disinfectant residuals. However, systems with high levels of biodegradable organic matter (BOM) levels need to maintain high disinfectant residuals to control microbial growth in distribution systems.

**Figure 4:**
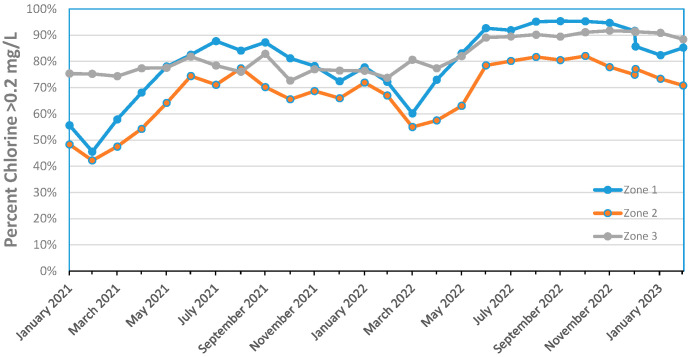
Percentage of chlorine residuals greater than 0.2 mg/L in three regions of an Australian water system.

Figure 4 shows the percentage of monthly distribution system samples containing greater than 0.2 mg/L free chlorine in various regions of an Australian water system. The percentage of samples containing >0.2 mg/L free chlorine was low (40%) in February of 2011 and generally increased throughout 2022 and early 2023. This trend of higher levels of chlorine in the system has continued since 2018. Zone 2 had more samples with lower levels of chlorine and perhaps benefited from the short-term supply of desalinated water rather than the usual unfiltered surface water supply.

Nutrient Levels. Microorganisms can grow if provided with organic and inorganic nutrients that promote replication. Typically, carbon is the growth-limiting nutrient in North American drinking water systems [67]; however, the concentration of nitrogen, phosphorus, and certain metals can affect microbial growth in drinking water distribution systems. For example, in certain locations, such as Finland, China, Norway, or Japan, low phosphorus levels could limit microbial growth in the distribution system [68,69].

**Figure 5:**
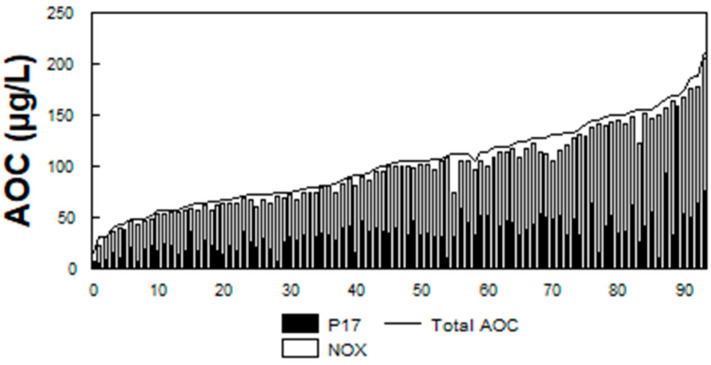
AOC levels in 94 North American water systems. From Volk and LeChevallier [29].

**Figure 6:**
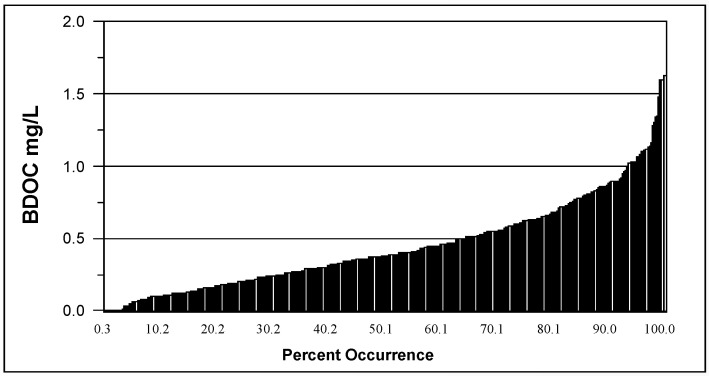
BDOC levels in 30 North American water systems. From Volk and LeChevallier [29].

The presence of biodegradable organic matter (BOM) in water has been associated with bacterial growth in drinking water [70]. BOM is commonly measured as assimilable organic carbon (AOC) or biodegradable dissolved organic carbon (BDOC). AOC is determined using a bioassay [71,72] and measures the microbial response to biodegradable materials in water. AOC levels (Figure 5) in 94 North American drinking water systems ranged from 20 to 214 µg/L, median 100 µg/L [29,46]. BDOC is the difference in the concentration of DOC before and after bacterial growth in a sample and measures the amount of nutrient readily available for bacterial growth [73]. Levels of BDOC in 30 North American water systems (Figure 6) ranged from 0 to 1.7 mg/L, with a median level of 0.38 mg/L [29].

**Figure 7:**
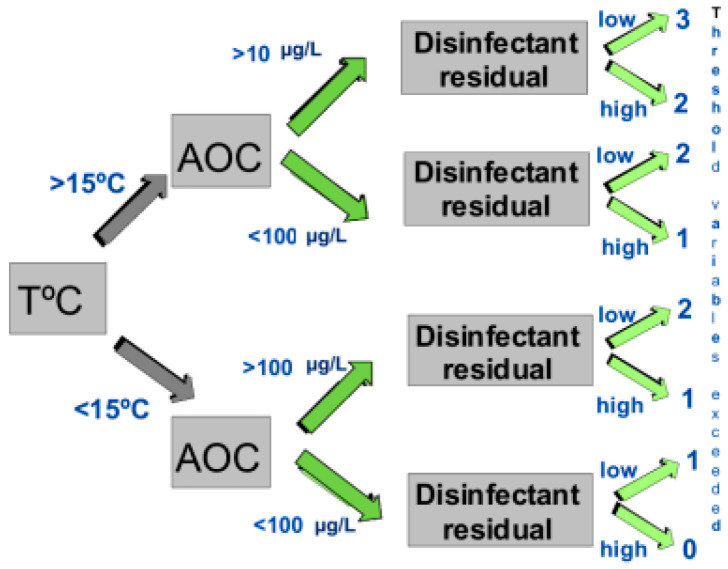
Decision tree for coliform occurrences in drinking water. Abbreviations: T °C, temperature in degrees Celsius, AOC, assimilable organic carbon. From Volk and LeChevallier [29].

On average, free chlorinated systems with AOC levels greater than 100 µg/L had 82% more coliform-positive samples, and the coliform densities were 19 times higher than in free chlorinated systems, with average AOC levels less than 99 µg/L [29]. However, high levels of AOC alone do not dictate the occurrence of coliform bacteria in drinking water but were only one factor. Figure 7 illustrates a decision tree that graphically depicts combinations of threshold values (including temperature, AOC levels, and disinfectant residuals), above which the probability of coliform occurrence is increased [29]. As more of the threshold values were exceeded, the probability of coliform occurrences is increased (Table 1). Systems that had water temperatures > 15 °C, AOC levels > 100 µg/L, and low (<0.2 mg/L free chlorine, or <0.5 mg/L chloramines) disinfectant residuals exceeded three of the threshold values and had an increased probability of coliform regrowth. Data summarized in Table 1 show that the frequency of coliform occurrence in 95 systems was less than 2% when no threshold criteria were exceeded and increased to 16% when all three criteria were exceeded. The magnitude of growth (the number of positive samples per event) also increased with a greater exceedance of threshold criteria. Similar models developed for specific systems have yielded higher predictive probabilities [74]. In systems that do not maintain a disinfectant residual, very low AOC levels (<10 µg/L) are required to minimize bacterial growth [71,72].

LeChevallier et al. [46] found that unfiltered water utilities experienced coliform bacterial growth at levels much higher than filtered systems. The authors attributed the difference to the presence of slowly degrading organic matter in the distribution system. Climate change could accelerate the growth of algal cells in source water supplies and the degradation of the cells in unfiltered drinking water systems could serve as an endogenous source of nutrients. Jjemba et al. [47] reported increases in chlorophyll A in the dead-end portions of reclaimed water distribution systems fed by unfiltered, open storage reservoirs. These reclaimed water networks were typically not flushed and the growth of opportunistic pathogens including *Legionella*, *Mycobacterium*, *Pseudomonas*, and free-living amoebae occurred in every system examined.

System Hydraulics–Stagnation. Whenever drinking water stagnates, microbial water quality degrades. Therefore, an increase in hydraulic residence time is an important factor related to microbial growth. With long residence times, chlorine residuals tend to dissipate, water temperatures rise, and bacterial levels increase. Increases in microbial levels due to growth have been related to distribution systems with a large number of storage tanks [46,75]. Ling et al. [75] reported that the bacterial community composition changed rapidly from the municipal supply following a 6-day stagnation, along with an increase in cell count from 10^3^ cells/mL to upwards of 7.8 × 10^5^ cells/mL. Changes in pipe diameter also impacted chlorine decay and microbial composition.

When water velocity slows, particularly in dead-end areas of the distribution system, sediments can precipitate, creating habitats for bacterial growth. Donlan and Pipes [76] showed that water velocity had an inverse relationship on biofilm counts. Increasing reservoir turnover, looping dead-end pipes, and flushing stagnant zones can help reduce hydraulic residence times. Occasionally, mistakenly closed valves can create artificial dead-end pipelines. A routine flushing and valve maintenance program is helpful for identifying closed valves and improving the circulation in the distribution system.

Reversal of water flows within the distribution system can shear biofilms and water hammer can dislodge tubercles from pipe surfaces. Opheim et al. [77] found that bacterial levels in an experimental pipe system increased 10-fold when flows were started and stopped. Larger releases of bacteria (sloughing) were noted when the system was exposed to physical and vibrational forces. Pipe vibrations caused by heavy traffic have been reported to cause the release of biofilm material [78].

Water age–related problems can be analyzed using existing water quality and operational information, use of tracer studies, and use of calibrated hydraulic models [79]. Existing water quality data such as temperature, disinfectant residual, and trihalomethanes (THMs) can provide valuable insight into locations that are experiencing water age problems. Alternatively, in systems with multiple sources with varying water quality characteristics (such as differences in water hardness or conductivity), these natural constituents can be used as a tracer. Mathematical models that represent the hydraulic behavior of the movement of water have been used to estimate water age in distribution systems [80]. Water quality models can be used in conjunction with hydraulic models to predict concentrations of chlorine, DBPs, and other constituents in a distribution system [81]. Several commercially available models can be used.

While identification of areas with water age-related problems can be straightforward, resolving these problem areas can be more complicated. Increasing turnover and reducing stratification in storage tanks through the design of inlet/outlet configurations, in-tank mixing, looping mains in low-flow areas of the distribution system, decreasing main diameters, or installing auto-flush valves are some of the options that could be examined when addressing water age issues (see: [79,82] and https://www.epa.gov/dwreginfo/drinking-water-distribution-system-tools-and-resources (accessed on 24 April 2024).

*Flushing, Cleaning of Pipes and Storage Tanks*. Flushing or other forms of main cleaning are recommended as one of many tools for controlling biofilm growth, removing accumulated sediment, restoring disinfectant residual, improving hydraulic capacity, and improving taste and odor in distribution systems [83,84]. Most loose deposits are readily removed from smooth pipes (such as PVC pipe or new ductile iron pipe) and slightly tuberculated pipe at velocities between 0.6 and 1.2 m/s (2 and 4 fps) [85]. With respect to tuberculated mains, higher flushing velocities are needed, but they tend to produce a diminishing performance for particle-removal performance for severely tuberculated mains. If the flushing objective is to scour and clean the pipe wall, high-velocity flushing (i.e., ≥1.5 m/s [5 fps]) is generally needed. However, flushing at excessively high velocities can damage pipe linings and result in decreased pressures upstream, and water may be wasted without obtaining any additional water quality benefit.

Unidirectional flushing (UDF) involves the sequential isolation of specific water mains to direct clean water (from mains previously flushed) at desired velocities and is the preferred method for removal of sediments. Conventional flushing (where hydrants are opened without isolation and regard to the upstream water quality) can simply move sediment from one location to another without actually flushing it out of the network. Many commercially available hydraulic models have modules to assist with unidirectional flushing so that water velocities can be attained while still maintaining adequate water pressure in the system. It is important that flushing programs be systematically and routinely implemented. In one study [86], coliform bacteria reappeared within 1 week after flushing a section of a distribution system, presumably because the organisms were growing in other parts of the pipe network.

An unpublished study of four locations in an Australian distribution system, showed some sites with high turbidity and ATP (Adenosine triphosphate—a measure of microbial abundance) and others with high iron and other metals (Table 2). The high heterotrophic plate count (HPC) and ATP levels are indicative of biofilms and biological activity and the high iron levels would make maintenance of a chlorine residual difficult (if not impossible, because the iron would react with free chlorine). Table 2 shows that after flushing, the levels of most of the measured constituents were reduced except for HPC counts at the Windmill site. The details of the flushing procedure were not provided in the report, but if these sites were just spot flushed (as opposed to a systematic flush of the system from the source to these locations), it is likely that some turbidity and HPC bacteria could have been washed into these areas from deposits upstream.

Distribution system maintenance, including cleaning and relining of corroded pipes and cleaning of accumulated sediments in storage tanks, is also important to reduce the habitats where bacteria grow in water systems. Lu et al. [87] used molecular methods (qPCR) to detect opportunistic pathogens in 87 sediments samples from 18 drinking water systems from ten U.S. states. *Mycobacterium* spp. Were detected in 88.9%, *Legionella* spp. In 66.7%, *P. aeruginosa* in 22.2%, and *Acanthamoeba* spp. In 38.9% of the samples. *N. fowleri* were not detected nor were most enteric pathogens (*Campylobacter jejuni*, *Escherichia coli* 0157:H7, *Salmonella enterica*, *Cryptosporidium parvum*, or *Giardia duodenalis*). Average genomic concentrations ranged between 2.5 × 10^2^ to 6.7 × 10^4^ cell equivalents [87]. In a follow-up study, Qin et al. [88] detected *L. pneumophila* genes in 25% of the sediment samples and 13% of the water samples from eight water storage tanks in three U.S. states. In this study, *P. aeruginosa* was not detected in the sediment samples but was found in 50% of the water samples. The authors concluded that sediments in storage tanks could serve as a habitat for growth of opportunistic pathogens and contribute these organisms to the overlaying bulk drinking water.

Pressure Management and Distribution System Integrity. Maintaining the physical integrity of the distribution system is critical to the microbial quality of the drinking water and protection of public health [89]. Physical integrity is defined as the ability of the distribution system to act as a physical barrier to prevent external contamination from affecting the quality of internal drinking water supply [89]. Physical integrity encompasses components including (1) pipelines, including mains and service lines, (2) fitting and appurtenances such as hydrants, valves, and meters, and (3) storage facilities and backflow prevention devices. Hydraulic integrity—the ability to provide a reliable water supply at an acceptable level of service—is closely linked to physical integrity as failure of water mains or storage tanks will lead to loss of hydraulic integrity. One of the most critical components of hydraulic integrity is the maintenance of adequate water pressure. Surges in water pressure (also known as water hammer) can create vibrations in pipes, possibly releasing biofilm material [90]. As already discussed, many water quality parameters change with the length of time in the distribution system—a factor directly linked to the hydraulic design of the system.

Breaches in the physical or hydraulic integrity of a distribution system—like main breaks or loss of water pressure—create the potential for contaminants to enter the pipe network. There is an opportunity for opportunistic pathogens to enter the system through these mechanisms. For example, *L. longbeachae* is known to occur in garden and potting soils and is particularly common in Australia [91,92]. Similarly, *M. avium* and other pathogenic mycobacteria can be found in natural water, soil, and dust. Thomson et al. [93] postulated that climate change could influence transmission of nontuberculous mycobacterial (NTM) infections in Queensland, Australia. Likewise, *P. aeruginosa* is commonly found in soil and water [94]. However, despite the presence of these opportunistic pathogens in water and soils that might enter the distribution system, no studies have proven this to be a pathway for contamination for these microbes. Although entry is possible, the focus of a water management plan is more on the management of growth of these microbes. Best practices for pressure management and sanitary repairs of main breaks are prudent steps to limit microbial risks [95,96,97].

Corrosion Control. Corrosion of iron pipes can influence the effectiveness of chlorine-based disinfectants for inactivation of biofilm bacteria [62,98]. Therefore, the choice of pipe material and the accumulation of corrosion products can dramatically impact the ability to control the effects of biofilms in drinking water systems. Corrosion of pipe surfaces provides not only protection from chlorine disinfectant residuals, but also a habitat for bacterial proliferation. In drinking water systems, the occurrence of coliform bacteria in corrosion tubercles on iron pipes has been reported by several investigators [86,99,100]. Laboratory studies showed that the densities of HPC and coliform group bacteria were ten times higher when grown on mild steel coupons than on non-corroded polycarbonate surfaces [101]. The increased surface area owing to tuberculation of the pipe walls, the concentration of organic substances within the tubercles, and the secretion of organic compounds by iron-using bacteria have been postulated as reasons why iron corrosion stimulates bacteria growth [84].

LeChevallier et al. [62] showed that the disinfection of biofilm on galvanized, copper, or PVC pipes was effective at 1 mg/L of free chlorine or monochloramine, but disinfection of organisms on iron pipes was ineffective even at free chlorine residuals as high as 5 mg/L for several weeks. In full-scale studies, systems that used a phosphate-based corrosion inhibitor had lower coliform levels due to regrowth than systems that did not practice corrosion control [46].

The pipe surface itself can influence the composition and activity of microbial biofilm populations [100]. Studies have shown that biofilms developed more quickly on iron pipe surfaces than on plastic polyvinyl chloride (PVC) pipes [98,101], and iron pipes support a more diverse microbial population than PVC pipes ([102]. *M. avium* levels were higher on iron and galvanized pipe than on PVC or copper surfaces. The resistance of *M. avium* to zinc may enhance its survival on galvanized pipe surfaces [103]. The effect of the combination of biodegradable organic matter, disinfectant type, and pipe composition on the survival of *M. avium* and HPC bacteria is shown in Table 3. The lowest level of biofilms occurred on copper pipe under low nutrient and free chlorine conditions.

Iron is an important nutrient for microorganisms involved in oxygen transfer, protein synthesis, and other essential metabolisms, and studies have shown that the presence of iron contributes to the growth of some opportunistic pathogens in drinking water. Bench-scale studies have demonstrated that iron concentrations of up to 1 mg/L could enhance *L. pneumophila* growth in tap water and field studies have observed similar positive correlations between *L. pneumophila* levels and iron concentrations [105].

Most utilities do not measure the corrosivity of their water on a daily basis, and variations in corrosion rates can be influenced by rainfall, changes in chemical coagulants, and temperature [106]. Moreover, many utilities associate corrosion control with management of lead and copper corrosion, ignoring the impact on iron corrosion, which comprises most of their system. In many cases where bacterial growth is occurring in the presence of an adequate disinfectant residual, an improvement in corrosion control can result in improved disinfection of biofilm bacteria [98,102,106,107]

## 5. Description of Specific Opportunistic Pathogens

### 5.1. Legionella pneumophila

Description. *Legionella* is an aerobic, non-encapsulated, non-spore forming, Gram-negative bacterium. The genus *Legionella* currently includes more than 61 species and 3 subspecies, only half of which have been associated with human infections [108,109]. Legionellae have a requirement for certain amino acids (cysteine) and iron, pointing to their role as intracellular parasites of free-living protozoa because the nutritional requirements for their growth would rarely be found in natural waters [110]. *L. pneumophila* has at least 18 serotypes. Legionnaires’ disease is an acute, sometimes fatal (5–30%), infection primarily exhibited as pneumonia with fever, cough, shortness of breath, and myalgias (i.e., soreness or aching of the muscles) [111]. Most Legionnaires’ disease cases (from 80 to 90 percent in Europe and the United States) are linked to *L. pneumophila* [112,113,114,115,116]; of these infections, *L. pneumophila* serogroup 1 is responsible for about 95% of the Legionnaires’ disease cases in the United States [110]. Collier et al. [7] estimated that hospital cost of a Legionnaires’ Disease case was over USD 37,000 and the overall annual healthcare cost is over USD 400 million.

Table 4 shows the *Legionella* species isolated in the European Union in 2020 and demonstrated the preponderance of *L. pneumophila* infections [117]. *L. pneumophila* serogroup 1 accounted for 95% of the 885 culture confirmed cases. The second most predominant species, *L. longbeachae*, is primarily transmitted through dust and soil [118]. *L. pneumophila* serogroup 1 grows better in humans and can evade human immune responses so that it survives better in human lungs [111]. The USEPA recently included *L. pneumophila* in the fifth list of the Candidate Contaminant List (CCL5) [119].

**Figure 8:**
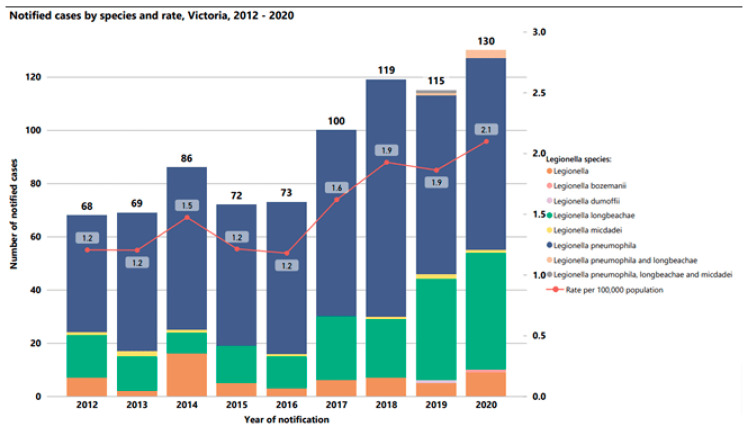
Legionella cases in Victoria, 2012 to 2020.

Similarly, the Victoria Department of Health reported increases in cases of Legionnaires’ Disease from 2012 to 2020 (Figure 8), where the incidence of disease per 100,000 people increased from 1.2 to 2.1. The health department reported that many of these cases were associated with cooling towers, but pneumonia cases associated with potting soil due to *L. longbeachae* were also a significant portion of the overall cases of Legionnaire’s Disease in the region. Figure 1 has already demonstrated the increase in drinking water outbreaks of Legionnaires’ Disease in the U.S since the year 2000.

**Figure 9:**
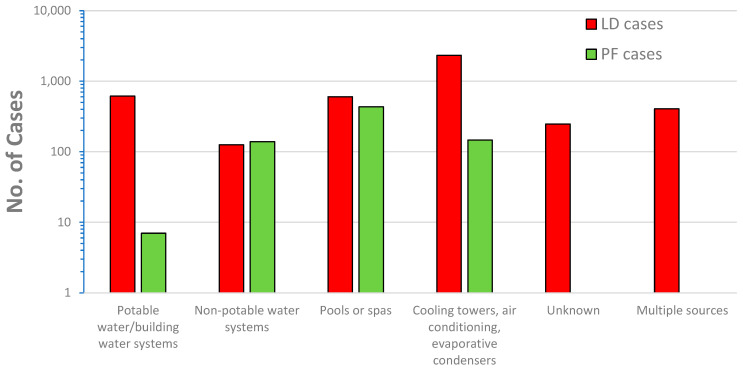
Comparison of Legionnaires’ Disease (LD) and Pontiac Fever (PF) Cases 2006–2017. Adapted from Hamilton et al. [120].

*Legionella* also causes a milder disease, called Pontiac fever, which includes fever, myalgias, chills, and headache, but, by definition, not pneumonia; most patients recover without treatment [111]. Hamilton et al. [120] conducted a review of Legionnaires’ Disease and Pontiac fever cases from 2006–2017 that included 136 outbreaks with 115 Legionnaires’ Disease outbreaks, 4 Pontiac fever outbreaks, and 17 mixed outbreaks of Legionnaires’ Disease and Pontiac fever. The researchers found Pontiac fever was mostly associated with non-potable water sources (i.e., fountains, pools, spas, cooling towers, etc.) and only 1% of the cases were associated with potable water (in building water systems) (Figure 9). All the cases of Pontiac fever were caused by *L. pneumophila* or mixtures of *L. pneumophila* and other *Legionella* species. Although Pontiac fever has been attributed to non-pneumophila species [110], it would appear, based on the data of Hamilton et al. [120], that the predominate risks are associated with *L. pneumophila*.

Health Risk. Legionellosis is most common among the elderly and those who are immunosuppressed. Incidence is higher in men and in people who smoke cigarettes. In 2016 in the United States, the incidence of legionellosis in men (2.31/100,000) was approximately 50 percent higher than in females (1.50/100 k) [111]. The risk of Legionnaires’ disease increases at approximately age 40 to 50 years [121,122] and cases in children are typically hospital-acquired and related to their immunodeficient status [123]. The mortality rate for Legionnaires disease is usually within the range of 5–10% but may be as high as 40–80% in untreated immuno-suppressed patients [8]. Hospital-acquired Legionnaires disease can have a death rate 25% [124].

In people with weakened immune systems, species other than *L. pneumophila* that are frequently isolated include: *L. micdadei*, *L. bozemanii*, *L. dumoffi*, and *L. longbeachae* [125,126]. These species cause infections primarily in patients undergoing cancer treatment or immunosuppression due to organ transplants. With few exceptions, reported clusters of pneumonia due to non-pneumophila *Legionella* species have been hospital acquired (nosocomial) [127]. Community outbreaks of pneumonia due to species other than *L. pneumophila* are uncommon, and most cases appear to be sporadic. Exceptions to this generalization are community outbreaks of *L. longbeachae*, which are typically associated with exposure to potting soil, not water systems [111,118]. According to Muder and Victor [127], there are no reported outbreaks of pneumonia due to non-pneumophila *Legionella* species that have been associated with large aerosol-generating devices, such as cooling towers.

A KWR and Berenschot 2021 report for the Dutch Ministry of Infrastructure and Water Management recommended a hybrid approach to monitoring for “all” Legionella species in hospitals and high-risk environments, and for only *L. pneumophila* in water distribution systems and buildings [128]. The report reasoned that it was primarily people with severely weakened immune systems that suffer from legionella strains other than *L. pneumophila*. The study found that there was no evidence to prove that monitoring other legionella strains was a good indicator of the presence of *L. pneumophila*. The report’s author, van der Wielen, stated that “different Legionella strains need different conditions to grow and the presence of any random *Legionella* species does not necessarily mean that the most pathogenic strains can also grow”. Monitoring for non-pneumophila species results in drastic measures to keep non-hazardous Legionella strains out of the systems, even though the health benefits are extremely limited. van der Lugt et al. [129] reported that a survey of 206 buildings in the Netherlands found 96.9% of the buildings were positive for non-pneumophila species of *Legionella* and that *L. pneumophila* was detected in only 3% of the samples. Similarly, Szczerba [130] reported finding *Legionella* spp. by PCR in distribution system water in 82% (433/528) of the samples in New York City, but *L. pneumophila* was detected by PCR in <1% (5/528) of the samples. Although there is on-going debate on the appropriate target for *Legionella* monitoring, some countries, like France, have implemented control measures both for cooling towers and hospital water networks that focus specifically on *L. pneumophila* [131].

**Figure 10:**
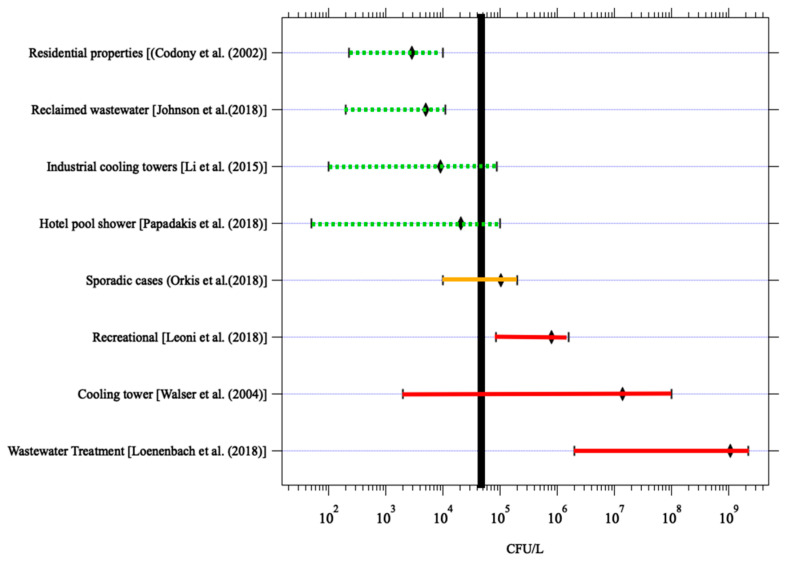
Concentration of culturable Legionella during outbreaks (red-orange) and routine monitoring (green). The black line is the 5 × 10^4^ cfu/L action level as a break between sporadic cases and outbreaks. From NASEM [111].

Quantitative microbial risk assessment (QMRA) is a mathematical modeling approach used to estimate the risk of infection and illness when a population is exposed to microorganisms in the environment. Hamilton et al. [132] conducted a QMRA for *L. pneumophila* exposure by various fixtures (i.e., showers, faucets, toilets) and usage rates (e.g., low-flow or conventional fixtures). The authors also calculated risks for clinically significant illness (CSI—a measure of legionellosis) and a probability of infection (an exposure but not necessarily resulting in illness). Concentrations of *L. pneumophila* in water were compared to various target goals like a 10^−4^ risk of infection/illness or a micro-DALY. Estimates of risk were based on annual exposure, but also on a per exposure risk. Because *Legionella* monitoring is currently conducted less frequently, it is often helpful to discuss the risk from a single sample value than an aggregate annual exposure. Of the exposures examined, showering with a conventional (high volume) shower head had the highest risk (due to the large number of respirable droplets) and the median 10^−6^ DALY per exposure was 3.64 cfu/mL for CSI [132]. This value is more conservative than the 50 cfu/mL trigger level (Figure 10) developed by the National Academy of Science, Engineering and Medicine (NASEM) panel based on analysis of outbreak and routine monitoring data [111]. The committee considered that *Legionella* concentrations of 50 cfu/mL should be considered an “action level”, that is a concentration high enough to warrant serious concern and trigger remediation.

A reverse QMRA was developed by Schoen and Ashbolt [133], in which the starting point is an infection in the human lung (acquired during showering) and then calculations are made “backward” to determine the concentration in water that would lead to an infection. With this analysis, they calculated the bulk water concentration of 3.5 × 10^3^ to 3.5 × 10^5^ cfu/mL would be required to deliver the required dose.

The objective of this discussion is not to try to develop a regulatory level for *L. pneumophila* in water, but to show that guidelines for a monitoring program can be developed to manage the organism in water systems at levels of low risk. The US Centers for Disease Control (CDC) developed guidance for management of *Legionella* in potable water (Table 5). They indicate that concentrations below 1.0 cfu/mL would indicate a well-controlled system. Sudden increases in concentrations or detection of *Legionella* in multiple samples would indicate that control measures were not working and that remedial actions should be taken. The “well controlled” trigger level is below the median 10^−6^ DALY per exposure of 3.64 cfu/mL for CSI developed by Hamilton et al. (2019) and the 10 cfu/mL limit is below the 50 cfu/L “action level” recommended by the NASEM panel [111]. The advantage of issuing this guideline is that it opens the door to enabling additional monitoring, which will provide a clearer picture of *Legionella* occurrence and levels in potable water supplies. This additional data will be helpful in eventually developing water management plans for *Legionella* in water supplies.

Occurrence in Distribution Systems. Despite nearly 50 years of *L. pneumophila* monitoring, there is surprisingly little data available for drinking water distribution systems. Traditionally, distribution systems were considered low risk because of their relatively cold water, but with the recent trend towards outbreaks in the potable water supply of buildings [13], distribution systems supplying the buildings have come under increased scrutiny. With the availability of easier methods for *Legionella* testing, there is also increased interest by utilities in knowing that they are adequately controlling *Legionella* in their distribution systems. That said, low levels of *L. pneumophila* may be able to break through treatment barriers entrapped in the cysts of free-living amoebae or inside protozoa hosts where they are protected from disinfection [51].

Analyzed by molecular methods, the DNA for *Legionella* species can frequently be detected at low levels in drinking water treatment plants and distribution systems. Wullings and van der Kooij [134] analyzed 16 surface water and 81 groundwater treatment plants in the Netherlands. They detected *Legionella* DNA by qPCR in 3 of 7 samples from anaerobic groundwater systems at a maximum concentration of 2.4 × 10^3^ cells/L; in 8 of 9 samples from aerobic groundwater systems ranging from 2.7 × 10^3^ to 2.5 × 10^4^ cells/L; and 33 of 34 samples of water collected from different treatment stages in 25 treatment plants with *Legionella* species at concentrations ranging from 1.1 × 10^3^ to 7.8 × 10^5^ cells/L. The highest *Legionella* concentration was observed in treated surface water with GAC filtration as the final treatment step (before post-disinfection). *L. pneumophila* was not detected in any of these water samples by qPCR and no culturable *Legionella* organisms were detected. These results are similar to a study in the New York city system, where *Legionella* species were detected by PCR in distribution system water in 82% (433/528) of the samples, but *L. pneumophila* was detected by PCR in <1% (5/528) [135]. Similarly, Gleason et al. [136] analyzed 58 sites for both first draw (representative of the faucet plumbing) and flushed cold water (3 min. flush) to represent distribution system water for a utility in New Jersey, USA. *Legionella* DNA markers were detected in 17.2% (10/58) of first the draw samples and in 15.5% (9/58) in flushed samples. However, Legionella spp. were detected by culture at low levels (0.5–1.6 cfu/mL in 4 of 58 (6.9%) of first draw samples and only one (1.7%) of the flushed samples. No *L. pneumophila* was detected by culture. Logan-Jackson and Rose [137] reported detection of *L. longbeachae*, *L. pneumophila*, and *L. micdadei* in the influent water pipes to an academic/research building at geometric mean concentration of 0.18, 0.26, and 0.2 genomic copies [GC]/100 mL, respectively.

Colbourne and Trew [138] reported that culturable *L. pneumophila* serotype 1, subgroup Olda, were detected in 2 of 62 taps located in buildings in 21 supply areas of the London distribution system. It is not clear from the study if the positive samples were representative of the distribution system or reflected the microbiota of the building plumbing. LeChevallier [34] analyzed 573 distribution system samples by the Legiolert method, and *L. pneumophila* was detected in 14 samples (2.4%): in 13 samples from free chlorinated systems (4.1% of 317 samples) and once in a chloraminated system (0.39% of 256 samples). None of the samples were positive for total coliform bacteria or *E. coli*, indicating that these indicator organisms are not valid predictors of *L. pneumophila* occurrence. For the 14 distribution system samples that were positive for *L. pneumophila*, individual sample concentrations ranged from 1 to 522 MPN/100 mL.

**Figure 11:**
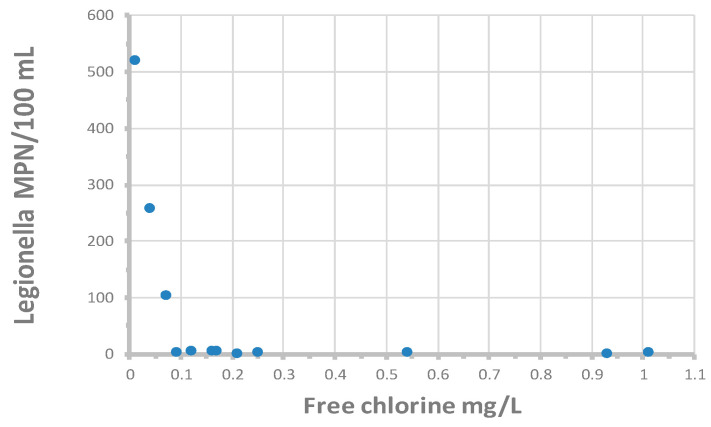
Relationship between *L. pneumophila* concentration and free chlorine residual. From LeChevallier [34].

Figure 11 shows the relationship between *L. pneumophila* cell densities and free chlorine residuals. All values greater than 10 MPN/100 mL occurred when free chlorine residuals were less than 0.1 mg/L. There was no relationship between *L. pneumophila* occurrence and free chlorine residuals when cell densities were less than 10 MPN/100 mL. The data suggests that, to prevent elevated concentrations of *L. pneumophila*, utilities should maintain at least a 0.1 mg/L in all parts of the distribution system. However, the data also show that low concentrations of *L. pneumophila* may occur even when free chlorine residuals are more than 1.0 mg/L. The one chloraminated sample that was positive demonstrated *L. pneumophila* at 4 MPN/100 mL, with a residual of 3 mg/L total chlorine. It is not clear why *L. pneumophila* is able to persist at high disinfectant residuals. Perhaps the cells were entrapped in the cysts of free-living amoebae or inside protozoa hosts where they are protected from disinfection [51] or protected within pieces of biofilm or suspended sediment.

**Figure 12:**
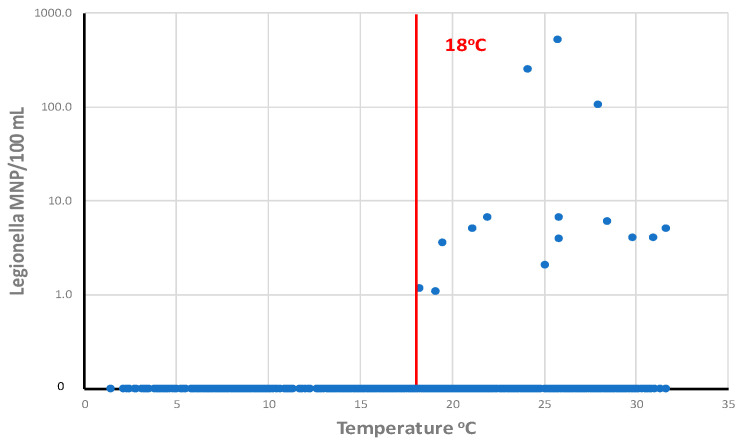
Relationship between temperature and concentration of *L. pneumophila*. From LeChevallier [34].

*Legionella* were most prevalent in drinking water distribution systems when water temperatures were greater than 18 °C (Figure 12). LeChevallier [34] analyzed a total of 1087 samples from 12 distribution systems and showed that the highest concentrations of *L. pneumophila* were observed in water samples with temperatures of 24–28 °C. *L. pneumophila* levels were the highest when disinfectant residuals were also low (<0.1 mg/L). However, it is impractical for most public water systems to effect major changes in water temperature in their distribution systems, but there are some practices that can be used by some utilities that can affect water temperature. For example, intakes can be positioned below the thermocline in some raw water supplies, so that the cooler source water can be withdrawn. Blending warm surface waters with cooler groundwater supplies can be conducted in some systems. Promoting water mixing and turnover in elevated storage tanks can prevent water stratification during warm weather and help control water temperature and disinfectant residual loss [139].

Elevated storage tanks may be prone to high water temperatures where water stratification may prevent mixing and subsequent loss of a disinfectant residual. Lu et al. [87] detected *Legionella* by qPCR in 66.7% of municipal drinking water storage tank sediments from 18 sites. Diverse *Legionella* spp., including *L. pneumophila*, *L. pneumophila* sg1, and *L. anisa*, were identified. A community outbreak of legionellosis in New Jersey that included cases of both Legionnaires’ disease and Pontiac fever was traced to a storage tank in the community water supply [140]. The storage tank had instances of low chlorine (<0.05 mg/L); *L. pneumophila* serogroup 1 was detected in 25% of the samples collected from mains prior to flushing the tank and in 50% of the samples during the tank flushing.

Legionella Risk Management. The principals outlined in the section on “Factors Influencing Growth of Microbes in Distribution Systems” apply to *Legionella* risk management, but this section will focus on specific data for *Legionella* disinfection and distribution system flushing.

Chemical Disinfection. Chemical disinfectants, particularly oxidizing agents such as chlorine, chlorine dioxide, chloramine, and ozone, are widely used to control *Legionella*. Other oxidizing agents including iodine, hydrogen peroxide, potassium permanganate, and bromine are rarely used and will not be discussed in this report as they are not typically used in distribution systems (for a review, see [111,141]). A difficulty in summarizing the disinfection literature for *Legionella* is characterizing the disinfection strategy used by the researcher. For example, studies could report the disinfectant concentration measured in mg/L and time of exposure measured in minutes (abbreviated “CT”) for planktonic cells, high disinfectant residuals used in response to an outbreak in a hospital or nursing home, or proactive or routine disinfection in the absence of detection of any *Legionella* bacteria.

Chlorine. Chlorine is the disinfectant most commonly used by water utilities [142]. Chlorine adversely affects the cell membrane, respiratory, enzymatic, and nucleic acids of microorganisms, leading to their inactivation [143]. During treatment, chlorine can be added to water as elemental chlorine (chlorine gas), sodium hypochlorite solution, or dry calcium hypochlorite. In water, chlorine exists as hypochlorous acid (HOCl) and hypochlorite ions (OCl^−^) where the hypochlorous acid predominates at pHs below 7.5 and is a more effective biocide.

Generally, maintenance of a free chlorine residual in potable water systems is effective for control of *Legionella* [143] but there are many situations where the bacteria can be shielded from the disinfectant (as in a biofilm or amoebae cyst) and therefore complete eradication of the organism is difficult. Planktonic *Legionella* resuspended in water were eliminated within 3 min by 2 mg/L free chlorine derived from sodium hypochlorite [144]. By comparison, *Legionella* spp. in protozoa cysts survived 25-fold more chlorine disinfectant after 18 h [52].

Laboratory studies conducted by Gião et al. [145] found that suspensions of 10^7^ cfu/mL of *L. pneumophila* (strain NCTC 12821) were not detected by cell culture after exposure to 0.7 mg/L of chlorine for 30 min at 20 °C, and at a dose of 1.2 mg/L, cultivability was lost after 10 min. At these high cell densities, the chlorine demand of the suspension was reported and chlorine values were adjusted for the chlorine demand. The researchers also reported that *Legionella* exposed to 1.2 mg/L of chlorine for 30 min and were subsequently co-cultured with *Acanthamoeba polyphaga* recovered their cultivability after 72 h. This repair of viable but non-culturable *Legionella* was also reported by Garcia et al. [146].

*Legionella* in the environment grows in the presence of host protozoa, but only a few studies have looked at disinfectant efficacy in co-culture with protozoa. Dupuy et al. [51] co-cultured amoeba and *L. pneumophila* and showed that free chlorine efficacy was better at higher temperatures, but the amount of chlorine required was different for the bacterium and its protozoan host (Table 6). An initial dose between 2 mg/L and 3 mg/L was applied, with a free chlorine residual of 1 mg/L at the end of the treatment. Chlorine was found to inactivate all three strains (M3, S2, and V1) of *Acanthamoeba* studied, both those infected with *L. pneumophila* and those not infected. At least a 3-log (99.9-percent) inactivation was obtained for all strains at a CT of approximately 80 min-mg/L at 30 °C, but this was reduced to 28 min-mg/L at 50 °C. For *L. pneumophila*, water temperature had a much greater effect on chlorine inactivation than did the co-culture conditions.

Amoebae cysts (the environmentally resistance resting stage) are much more resistant to disinfection than the free living trophozoites. De Jonckheere and Van de Voorde [147] reported a CT_99%_ of 1260 to 6480 mg-min/L for inactivation of *Acanthamoeba* cysts at pH 7.35 and 25 °C. Kilvington and Price [52] reported *Acanthamoeba polyphaga* cysts required high levels of free chlorine (75 mg/L for 18 h at 25 °C) to prevent excystation.

Maintenance of a continuous chlorine residual can create an environment where amplification of *Legionella* is unfavorable. The Allegheny County (Pittsburgh) Health Department specifies that potable water, from entering a building through to all distal outlets (e.g., faucets, shower heads), should be maintained at least 0.3 mg/L free residual chlorine [148]. The Pennsylvania Department of Environmental Protection has proposed establishing a minimum disinfectant residual requirement of 0.2 mg/L throughout drinking water distribution systems, primarily as a means for controlling *Legionella* outbreaks [149,150]. The USEPA is currently revising the residual disinfectant requirements of the Surface Water Treatment Rule and will likely require a minimum disinfectant residual of 0.2 mg/L as a means of limiting *Legionella* occurrences in distribution systems [12]. As shown previously in Figure 12, LeChevallier (2019b) reported that levels of *L. pneumophila* > 1.0 MPN/mL could be limited by maintaining a free chlorine residual of at least 0.1 mg/L.

Chlorine dioxide. Unlike free chlorine, chlorine dioxide does not hydrolyze when it enters water; it remains a dissolved gas in solution. As a neutral compound, it can easily diffuse through cell membranes of microorganisms where it disrupts protein synthesis. It has been found to be effective in penetrating biofilms as compared to chlorine [143,151,152]. Generally, chlorine dioxide is a more effective residual disinfectant for inactivating bacteria, viruses, and protozoan pathogens. Chlorine dioxide is an effective biocide over a wide pH range, but it is affected by temperature [152]. It is typically generated onsite for immediate use by slowly adding a strong acid (e.g., chlorine or sulfuric acid) to a sodium chloride solution. Despite the effectiveness of chlorine dioxide, it is not commonly used as a disinfectant in the distribution system due to the toxicity of the disinfectant and some of its by-products [153] as well as the potential for objectionable odors [154].

Loret et al. [155] compared the performance of chlorine dioxide and free chorine for control of *Legionella* grown in biofilms in a pilot-scale pipe loop system incubated at 30 °C. Chlorine dioxide was applied at a dose of 0.5 mg/L. *Legionella* populations decreased to undetected levels (<500 cfu/L) within six days of treatment for all disinfectants. Walker et al. [151] reported total elimination of *Legionella* in a hospital water system after treatment with 50–80 mg/L chlorine dioxide. The efficacy of chlorine dioxide for inactivation of *Legionella*, *Hartmanella*, and *Acanthamoeba* is shown in Table 7.

Monochloramine. Monochloramine is commonly used as an alternative to free chlorine to achieve compliance with requirements for disinfection by-products [153]; however, the disinfectant has shown to have advantages for control of *Legionella*. Monochloramine is formed by adding free chlorine in a solution of ammonium chloride at a chlorine to nitrogen molar ratio of 0.5 (pH 8.5). Also formed during the process are dichloramine and trichloramine. However, with the correct chlorine and ammonia mixing and alkaline pHs, monochloramine is expected to dominate [145]. The three species of chloramines are commonly referred to as “combined” chlorine. Disinfection with chloramine gained traction because the disinfectant is more stable in the system, minimizes the formation of disinfection by-products, and can penetrate biofilms better than free chlorine [65,158,159]. Monochloramine has a lower chlorinous odor threshold than free chlorine [160] but has a much lower disinfection efficacy than free chlorine [161] and requires a much longer contact time if used as a primary disinfectant. Relative to free chlorine (Table 6), the CT values for *Legionella* disinfection for monochloramine are higher (Table 8), suggesting that higher disinfectant doses or longer contact times are needed to achieve the same level of inactivation.

Chloramines appear to be more effective than free chlorine in reducing the risks from *Legionella*. Kool et al. [162] examined 32 hospital-acquired (nosocomial) outbreaks of Legionnaires’ disease from 1979 to 1997 where drinking water was implicated and tabulated the characteristics of the hospital (i.e., size, transplant program) and the primary disinfectant treatment, disinfectant residual, water source, community size, and pH of the water. The researchers found that the odds of a nosocomial Legionella outbreak were 10.2 (95 percent CI 1.4–460) times higher in systems that maintained free chlorine than in those using a chloramine residual. Heffelfinger et al. [163] reported that 25 percent (38) of 152 hospitals surveyed had reported cases or outbreaks of hospital-acquired Legionnaires’ disease during the period 1989–1998. However, hospitals supplied with drinking water disinfected with monochloramine were less likely (OR = 0.20, 95 percent CI 0.07–0.56) to have hospital-acquired Legionnaires’ disease than other hospitals. Suspensions of *L. pneumophila* were more sensitive to monochloramine disinfection, with a 99 percent level of inactivation when exposed to 1.0 mg of monochloramine per liter for 15 min compared with the 37-min contact time required for *E. coli* inactivation under similar conditions. Donlan et al. [164] reported that monochloramine was significantly more effective than free chlorine at eradicating laboratory-grown biofilms of *L. pneumophila*. Lin et al. [152] reported that in a hospital in Washington, DC, a monochloramine concentration of 0.31 mg/L was effective in reducing *Legionella* counts in the building plumbing system. Loret et al. [155] found that planktonic *Legionella* decreased to undetectable levels after being dosed with 0.5 mg/L monochloramine in a model potable water pipe system for three days and remained undetectable for the remainder of the one-month experiment. There was no viable *Legionella* in the biofilm after six days of treatment.

Flannery et al. [165] showed a 93 percent reduction in the occurrence of *Legionella* in building plumbing systems in San Francisco after the utility converted from free chlorine to chloramines. Amoebae at sampled sites were associated with *Legionella* colonization predominately when chlorine was used for residual disinfection. *Legionella* were cultured from 61 (36 percent) of 169 samples in which amoebae were present versus 291 (24 percent) of 1236 samples without amoebae (*p* = 0.01). After conversion to monochloramine, *Legionella* were found in 1 (1 percent) of 78 samples containing amoebae and 8 (1 percent) of 866 samples without amoebae (*p* = 0.75). The prevalence of amoebae decreased from 169 (12 percent) of 1405 samples when chlorine was the residual disinfectant to 78 (8 percent) of 944 samples collected after conversion to monochloramine (*p* = 0.006). *Legionella* occurrence in Pinellas County, FL, was reduced when the system converted from chlorine to monochloramine disinfection [166]. Water samples were collected from 96 buildings (public buildings and individual homes) for a 4-month period when chlorine was the primary disinfectant and from the same sampling sites for a 4-month period after monochloramine was introduced into the municipal water system. When free chlorine was used, 20 percent of the buildings were colonized with *Legionella* in at least one sampling site. *Legionella* colonization was reduced by 69 percent within a month after chloramination. Monochloramine appeared to be more effective in reducing *Legionella* in hotels and single-family homes than in county government buildings, perhaps because of more consistent water usage. Holsinger et al. [167] analyzed 184 legionellosis outbreaks in the United States from 2001 to 2017 and found that 85% (*n* = 127) were associated with building water systems receiving chlorinated water while 10% (*n* = 15) received chloraminated water (the remaining outbreaks lacked data on the type of disinfectant used).

The effectiveness of monochloramine is generally thought to be due to the ability of the disinfectant to penetrate biofilms and inactivate the bacteria [65,158,159,164]. Lee et al. [159] and Pressman et al. [65] used microelectrodes to investigate the penetration of chlorine, monochloramine, oxygen, and free ammonia in nitrifying biofilms. Although the research demonstrated that monochloramine had greater penetration, the authors found this penetration did not necessarily translate to immediate loss of viability. Johnson et al. [168] suggested a different mechanism by which monochloramine could be effective for *Legionella* control. They found that amoebae in five free chlorinated reclaimed water systems were mostly (50–95%) in the active trophozoite phase; however, in the chloraminated system, 87% of the mesophilic amoebae and 66% of the thermophilic amoebae were in the cyst phase. They hypothesized that that the penetration of chloramines into the biofilm might trigger the amoebae to form cysts rather than outright kill the protozoa. In the water environment, *L. pneumophila* amplifies in the vacuoles of infected protozoa and this amplification occurs only in the trophozoite stage. Schoen and Ashbolt [133] reported a critical density of free-living protozoan hosts in biofilm required to propagate sufficient *Legionella* to cause infection, and infection was 3.1 × 10^4^ to 7.8 × 10^7^ host/cm^2^, suggesting that it may be possible to manage *Legionella* risk by limiting the free-living trophozoite population. Additional research is needed to evaluate this hypothesis but understanding the relationship between the protozoan host life stage, the *Legionella* bacterium, and the disinfectant is critical to designing control strategies for water systems.

Ozone. Ozone attacks unsaturated bonds of aldehydes, ketones, and carbonyl compounds [169] and can participate in electrophilic reactions with aromatic compounds and neutrophilic reactions with many cellular components (i.e., fatty acids, carbohydrates, amino acids, proteins, and nucleic acids). These reactions collectively affect the cytoplasmic membrane of bacterial cells and the protein structure as well as DNA. However, because ozone does not form a stable residual and decomposes rapidly in water, it is not typically used for pipeline or building plumbing systems, but primarily primary disinfection. It will be only briefly reviewed here.

Muraca et al. [170] applied ozone at 0.5 mg/L to achieve 5 log inactivation of *L. pneumophila* in 5 h using an initial concentration of 10^7^ cfu/mL (Table 9). Ozone efficacy was not impacted by temperature (25 °C versus 43 °C) or turbidity although the level of turbidity was not quantified. Based on Domingue et al. [171], the efficacy of ozone was not affected by pH or temperature. CT values from Muraca et al. [170] for reducing *Legionella* were much higher than those reported by others, possibly because they dosed their system to maintain a very high ozone residual of 1 to 2 mg/L. Lower CT values are required to control both *Naegleria* and *Acanthamoeba* cysts with ozone at 20–25 °C (Table 9). Overall, ozone is more effective than chlorine dioxide, which was in turn more effective than chlorine (i.e., O_3_ > ClO_2_ > Cl_2_) for primary treatment.

Ultraviolet Irradiation. UV does not kill microorganisms but rather damages their DNA, which prevents them from replicating, which in turn prevents infectivity. Similar to the CT concept, UV intensity (mW-s/cm^2^) times the exposure time (s), commonly referred to as fluence (mJ/cm^2^), describes UV disinfection capability. Fluence represents the energy per unit area falling onto a surface. Maximum efficacy with UV is attained at 254 nm [143] but turbidity, natural organic matter content, and particulate matter can significantly affect UV disinfection capability.

Hijnen et al. [174] reported a log reduction of *Acanthamoeba* with 40 mJ/cm^2^ and three log inactivation of various *Acanthamoeba* species and *V. vermiformis* were achieved with fluences of 23 to 100 mJ/cm^2^; the higher levels being required for cyst inactivation (Table 10). Overall, inactivation of *Acanthamoeba* and *Vermamoeba vermiformis* required higher levels of UV compared to *Giardia* or *Cryptosporidium* [175]. Because UV does not provide a residual, it is typically combined with a chemical disinfectant to effectively control *Legionella*.

All *Legionella* isolates tested by Cervero-Aragó et al. [176] required 5–6 mJ/cm^2^ UV fluence to inactive 4 logs. However, a higher fluence was required when co-cultured with amoeba (Table 10). UV irradiation at 30 mJ/cm^2^ reduced *L. pneumophila* by 5 log units in 20 min [170] although the very high concentrations of the bacteria could have affected the UV adsorption of the suspension (Table 10). Schwartz et al. [177] reported survival of *Legionella* in biofilms formed on polyethylene, PVC, and stainless-steel coupons following disinfection with UV, but no *Legionella* was detected on copper coupons. UV disinfection was not affected by temperature and is independent of pH [178]. *Legionella* inactivation requires slightly higher doses when the bacteria are exposed to light repair and have a similar level of inactivation with both low pressure and medium pressure lamps (Table 11).

Pipe Flushing/Cleaning. As mentioned previously, it is important that public water systems have a routine program for systematically flushing and cleaning the distribution system, as over time post precipitation of treatment chemicals, fine silts, and corrosion products can form sediments within pipelines and storage tanks that promote bacterial growth. Implementation of a “uni-directional” flushing program is recommended, where hydrants are opened near the treatment plant, and the water is flushed systematically away from the plant towards the ends of the system to avoid recirculating unflushed pipes into the cleaned sections of the system. Application of a hydraulic model is useful to ensure that adequate water pressure is maintained while achieving the targeted flushing velocity (>5 ft/s) [85,181].

**Figure 13:**
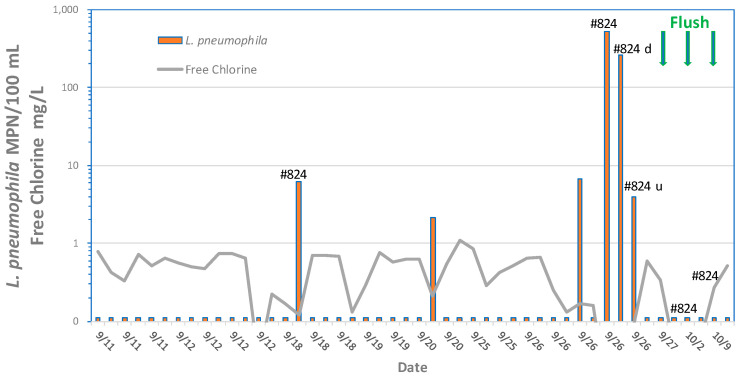
Impact of distribution system flushing at site 824 to control the occurrence of culturable *L. pneumophila*. Symbols: d, downstream f site 824; u, upstream of site 824. From LeChevallier [34].

Figure 13 shows the experience of a utility that detected *L. pneumophila* in an area of the distribution system that was under construction. The area had plans for several hundred homes, but at the time only 73 houses had been built [34,182]. The area is served by a 12 inch (30.5 cm) main, so detention times were very long. The free chlorine residual on September 18 in the site 824 sample was 0.12 mg/L, which was low but not unusual for the system that normally maintained an average of 0.4 mg/L within the system. On September 26, four of the nine samples collected that day were positive for *L. pneumophila*, with some of the concentrations as high as 522 MPN/100 mL. The free chlorine concentration in the area of site 824 ranged between 0.01 and 0.09 mg/L. In addition to resampling the original site, sites upstream and downstream were analyzed (Figure 13); all three were positive for *L. pneumophila*. Because of the low chlorine residuals, the utility flushed the area around site 824 on September 27 and again on October 2. The result of all the flushing was that free chlorine residuals increased to levels between 0.27 and 0.51 mg/L and samples were no longer positive for *L. pneumophila.* An automatic flush valve was installed on October 5, with a flush rate 27,216 L per day (7200 GPD) and free chlorine residuals increased to levels above 0.5 mg/L. *L. pneumophila* has not been detected at this site in more than three years of subsequent testing. The utility also redirected some of their capital funds to improve the consistency of disinfectant residuals across the distribution system and to improve turnover within storage tanks and reduce stagnation, and resulted in lowered DBP levels [183].

In the unpublished flushing study (Table 2), *Legionella* species DNA was detected at the Warrens Brook Rd site before flushing but was negative after flushing. No *L. pneumophila* DNA was detected in any of the samples. In this case, flushing was effective for removing the bacteria from the system.

#### *Mycobacterium* *avium*

Description. Currently, there are over 180 recognized species of *Mycobacterium*, the only genus in the family *Mycobacteriaceae* [184]. Organisms belonging to this genus are diverse with respect to their ability to cause disease in humans; some are strict pathogens (i.e., *M. tuberculosis* or *M. leprae*), while others are opportunistic pathogens (like *M. avium*) or nonpathogenic [185]. Bacteria in this genus have cell walls with Gram-positive and Gram-negative features, and acid-fast staining is used to emphasize their resistance to acids. The cell walls of mycobacteria are very thick and consist of four layers, including peptidoglycan and mycolic acids. The cell wall composition renders mycobacteria hydrophobic, and as a result these bacteria tend to grow in aggregates, forming difficult-to-eliminate biofilms, and adhere to surfaces in moist environments, such as the insides of plumbing in buildings [184]. The hydrophobic nature of the cell wall and the tendency to form biofilms and clumps make mycobacteria very resistant to disinfectants [186].

Environmental mycobacteria can be found in diverse environments and most appear to have a saprophytic lifestyle [187]. However, some can infect animals, birds, and humans, and have evolved mechanisms to evade and grow within host cells. Environmental mycobacteria can be found in soil, dust, and water, including natural water sources (such as lakes, rivers, and streams) and municipal water sources [184]. Environmental mycobacteria include both slow growing (i.e., colony formation requires 7 days or more; often 14–21 days) and rapidly growing (i.e., colony formation in less than 7 days) species. In fact, based upon differences in 16S rRNA gene sequences, the slowly and rapidly growing mycobacteria could be split into two different genera [188].

Environmental mycobacteria also have extraordinary starvation survival [189], persisting despite low nutrient levels in tap water. *M. intracellulare* persisted with only one log loss of viability after 1.4 years in deionized sterile water [57]. Carson et al. [190] reported growth of *M. chelonae* in commercial distilled water to levels of 10^5^ to 10^6^ cells/mL, and levels were maintained over a 1-year period. The tolerance of environmental mycobacteria to temperature extremes results in contamination from hot tap water and spas to ice machines [191].

The clinical terminology “Nontuberculous Mycobacteria” (or NTM) refers to infections caused by mycobacteria other than *M. tuberculosis* (the cause of tuberculosis) and *M. leprae* (the cause of leprosy). In clinical parlance, NTM are also referred to as atypical mycobacteria or mycobacteria other than tuberculosis (MOTT). However, for environmental applications, the terminology is inappropriate and should not be used because all environmental mycobacteria are “Nontuberculous Mycobacteria” (*M. tuberculosis* and *M. leprae* do not grow in the environment). Using the term “NTM” to refer to environmental isolates imparts an “air of importance” to the results when in fact the analysis (which usually is a molecular test) is simply a test for the genus *Mycobacterium*. It would be more accurate to call the results of such a test as “mycobacteria or members of the genus *Mycobacterium*”, but measuring NTMs sounds so much more important. The terminology “NTM” will not be used in this report unless it refers to clinical infections.

The USEPA recently included *M. avium* and *M. abscessus* in the fifth list of the Candidate Contaminant List (CCL5) [119] to highlight the potential health risks of pathogenic mycobacteria in water supplies. However, it was surprising that the USEPA also did not include other pathogenic mycobacteria. EPA scientist M.J. Donohue [5,6] reviewed clinical laboratory reports and found that U.S. clinical cases of mycobacteria increased from 8.2 per 100,000 persons in 1994 to 16 per 100,000 persons in 2014. Changes in mycobacteria diversity were observed in complex/groups known to be clinically significant. Between 1994 and 2014, the rate implicating *M. abscesses-chelonae* group (MABS) and *M. avium* complex (MAC) increased by 322% and 149%, respectively. In addition, the two listed CCL5 mycobacteria, *M. fortuitum*, *M. gordonae*, *M. mucogenicum*, *M. chelonae*, *M. kansasii*, and *M. xenopi*, all had significant rates of clinical illness. King et al. [192] detected *M. avium* and *M. intracellulare* in 36% of treated drinking water samples examined. It would be more helpful if researchers examined this group of pathogenic mycobacteria to focus research and public health protection on a more identifiable and actionable group of opportunistic pathogens than the nondescript “NTM” designation.

Disease /Health Risk. Collier et al. [7] estimated the overall annual healthcare cost in the US for NTM infections at USD 1.5 billion, including emergency department visits and hospitalization. The researchers estimated that 72% (range 39–94%) were acquired from waterborne sources and resulted in nearly 69,000 cases per year. NTM infections reflected nearly half of the overall healthcare cost from all waterborne infections (total USD 3.3 billion). Gerdes et al. [193] reported that NTM infections from drinking water accounted for 75% of the USD 1.4 billion in direct health care costs for emergency room visits and hospitalizations.

NTM infections occur in persons with underlying lung disease including cystic fibrosis, chronic obstructive pulmonary disease (COPD), emphysema, bronchiectasis, prior pulmonary infections, and lung cancer, or depressed immune systems including AIDS, leukemia, rheumatoid arthritis, and immunodeficiency from stem cell and organ transplants [184,194]. Besides pulmonary infections, mycobacteria infections can occur in skin and soft tissue, typically following surgery, trauma, injections, or visits to nail salons or tattoos; device-associated infections (e.g., prosthetic, intravenous needles, pacemaker, cosmetic implants, etc.); lymph nodes in the neck (associated with tooth eruption in children); and blood or other usually sterile locations in the body due to invasive medical devices or procedures (CDC 2023). NTM lung infections can affect elderly white postmenopausal females described as “Lady Windermere syndrome” [194]. It is important to note that while mycobacteria may be present in the hospital water supply, it is primarily contamination by non-aseptic procedures (e.g., carryover from ice, surgical items, or heater-cooler devices) that is the cause of the infection. Water is only incidental to these infections. In sub-tropical climates like parts of Australia, infections by *M. ulcerans* can be transmitted by mosquitoes [195]. For the most part, infections by environmental mycobacteria are not transmitted person-to-person [184].

**Figure 14:**
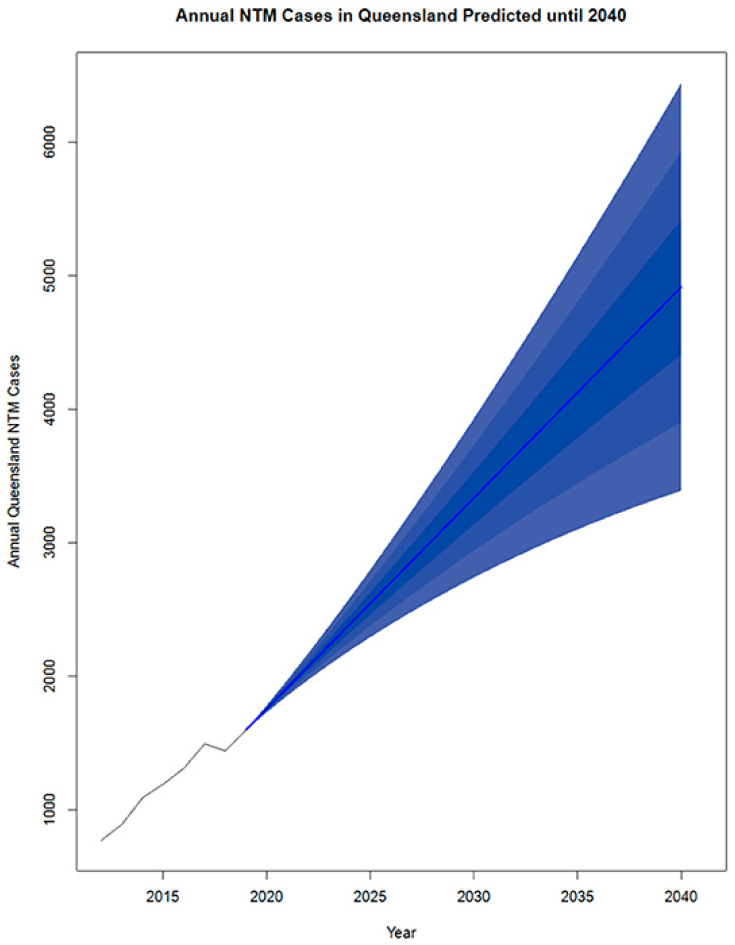
Projected NTM cases in Queensland, Australia from 2020 to 2040. From Ratnatunga et al. [194].

Mycobacteria infections have been a notifiable disease in Queensland (QLD), Australia, since the commencement of the tuberculosis (TB) control program in the 1960s [196]. Clinical cases of MAC disease were 0.63 cases/100,000 in 1985 and increased to 2.2 cases/100,000 in 1999. NTM infections increased to 13.6 cases/100,000 from 1999 to 2005. In total, 1171 NTM isolates were reported in 2016 [196]. More NTM infections occurred in males in 1999 but the pattern changed and predominated in females in 2005, particularly in the elderly. Similarly, an increase in NTM disease has also been seen in the Northern Territory (NT), Australia, from 1989 to 1997 [197]. Subsequent investigation showed MAC and MABS were present in household and municipal water sources and shower aerosols in homes [198,199]. Projections show that NTM cases could more than triple between 2020 and 2040 (up to 6446 cases per year) (Figure 14).

Although research has detected environmental mycobacteria in drinking water and water system biofilms, suggesting that contact with drinking water might be one source of pulmonary infections [200,201], substantial knowledge gaps, including the lack of risk assessment models, difficulty in evaluating mechanisms of exposure, and host-specific factors that influence susceptibility, have made it difficult to link NTM infections to drinking water and develop mitigation strategies [202]. Rusin et al. [203] estimated the oral infectious dose for *M. avium* for mice is 10^4^–10^7^ but determining the drinking water exposure for humans is complex. Tolofari et al. [204] suggested that the risks of *M. avium* in drinking water to be low (the annual DALY of MAC pulmonary disease was 4.74 × 10^−12^).

Occurrence in Distribution Systems. It is no surprise that mycobacteria are commonly found in drinking water biofilms, as the genus has been described as “early biofilm colonizer” [205]. Revetta et al. [206] reported that *Mycobacterium*-like phylotypes were the most predominant populations (>27%) in subsequent months of biofilm colonization in a drinking water pipe loop system. Using molecular methods that target the genus *Mycobacterium*, many researchers commonly report the frequent presence of mycobacteria in drinking water supplies [202]. Therefore, it is necessary to look beyond the studies of “NTM” occurrence and focus on specific opportunistic species.

**Figure 15:**
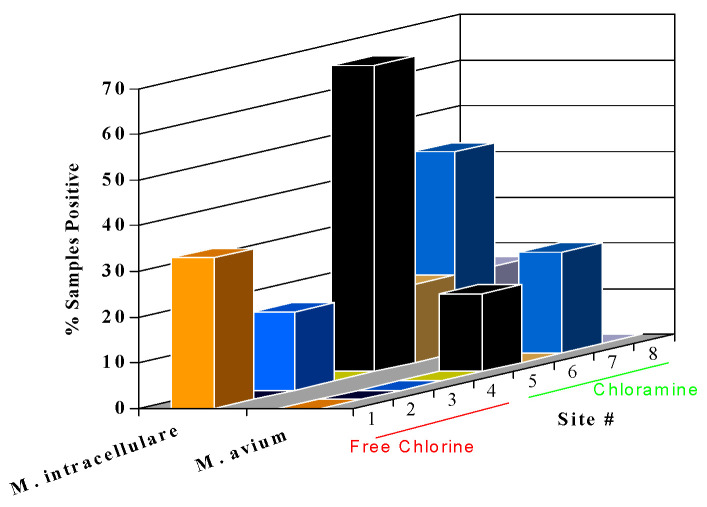
Occurrence of *M. avium* and *M. intracellulare* in distribution system biofilm samples (N = 55). Site # - site number. From Falkinham et al. [50].

Falkinham et al. [50] examined eight well characterized drinking-water systems, including surface and groundwater supplies and varying levels of AOC and BDOC. Samples were collected monthly for 18 consecutive months from the raw water, plant/well effluent, a distribution system mid-point, and a dead-end site. *M. avium* and *M. intracellulare* were frequently isolated by culture from drinking-water biofilm samples (Figure 15). The data showed that *M. avium* levels were reduced by conventional water treatment but increased due to regrowth in the distribution system. Increases in *M. avium* levels in drinking water correlated with levels of AOC (*r*^2^ = 0.65, *p* = 0.029) and BDOC (*r*^2^ = 0.64, *p* = 0.031) [50].

Pryor et al. [207] detected *M. avium* only once, but 21 types of mycobacteria were detected, with *M. intracellulare* most frequently detected in a groundwater fed distribution system. Mycobacteria detection frequency increased when the utility switched from free chlorine to chloramines (from 19.1% to 42.2%) in showerhead samples, where *M. gordonae* and *M. intracellulare* were the two predominant species. Glover et al. [208] recovered *M. avium* by culture from 70% of hospitals, 9% of dwellings, and 15% of reservoirs in a Los Angeles water system.

A survey conducted by Hilborn et al. [209] examined 56 tap samples originating from two different drinking water treatment plants and found that 54% of point-of-use filter samples were positive for *M. avium* using the culture method. The study found three different *M. avium* genotypes persisted at three different taps for 18–26 months. Similarly, King et al. [192] compared 25 treated drinking water utilities, and while *M. avium* and *M. intracellulare* were not detected in treated groundwater samples (0/3), they were detected in 24% (6/25) of the treated surface water samples by qPCR, but not by culture. *M. avium* and *M. intracellulare* average concentrations at the distribution entry point were 2.1 × 10^3^ and 8.0 × 10^2^ genomic units/L, respectively. Detection frequencies were higher in distribution system entry points of chloramine-treated surface water samples than for chlorine-treated surface water supplies (*M. avium* 33% (3/9) and *M. intracellulare* 44% (4/9) in chloraminated surface water, compared to *M. avium* 23% (3/13) and *M. intracellulare* 23% (3/13) for chlorinated surface water). Wang et al. [210] examined two chloraminated systems where samples were collected before and after flushing the taps for 3 min. *M. avium* was detected by qPCR in the first draw samples but not in the flushed samples, indicating that the bacteria were primarily colonizing the faucet plumbing.

Covert et al. [211] conducted the first US survey of mycobacteria in the US, examining 105 tap samples by culture methods, of which <1% (1/105) were positive for *M. avium* and 5% (5/105) were positive for *M. intracellulare*. Pfaller et al. [212] conducted a second survey, collecting water samples from 40 sites from different regions of the US. These samples represented three water types: groundwater disinfected with chlorine and surface water disinfected with chlorine or monochloramine. Samples were collected from private residences and commercial buildings after a 15 s flush, so they reflected more the building plumbing than the distribution system. *M. avium* and *M. intracellulare* were both measured by culture and qPCR. *M. avium* and *M. intracellulare* were detected by qPCR in 25% (73/292) and 35% (102/292) of samples, respectively. The mean concentrations of *M. avium* and *M. intracellulare* were 2.8 × 10^3^ and 4.0 × 10^3^ genomic units (GU) per liter. The Northeast region of the US had the highest sample positivity rate for both *M. avium* and *M. intracellulare*. The highest *M. avium* concentrations were observed in the surface water treated with chloramine. The authors concluded that geographic location and source water/disinfectant type significantly influence *M. avium* and *M. intracellulare* occurrence rates.

Whiley et al. [213] examined the occurrence of *M. avium* complex using qPCR in two potable water distribution pipelines in Australia. Distribution System 1 (DS1) was treated with coagulation, flocculation, sedimentation, full-flow micro-filtration, and disinfection with chlorine whereas water within Distribution System 2 (DS2) was treated with coagulation, flocculation, sedimentation, sand filtration, disinfection with ultraviolet light, and chloramine. *M. avium* complex levels in DS1 were the highest (30,000 copies/mL) in the summer (February), particularly at sites where the chlorine residual was <0.1 mg/L. In DS2, where chloramines were used, *M. avium* complex levels were significantly higher, particularly in dead-end areas of the system remote from the treatment plant.

Mycobacteria Risk Management. The goal of water treatment is to reduce the overall public health risk to the lowest level possible while balancing various competing risks (i.e., chemical versus microbial). Available research suggests that some water treatment processes (e.g., chloramination) may have some benefits for managing *Legionella* risk but could increase the frequency of detection of some mycobacteria species. The exact nature or quantification of these risk trade-offs is currently unknown, and the exact role of drinking water exposure is uncertain for mycobacteria risks.

Disinfection. The high concentration of mycolic acid and the hydrophobic surface characteristics of mycobacteria are also primarily responsible for the high resistance of the group to chemical disinfection. In fact, mycobacteria and other mycolic acid-producing genera (*Nocardia*, *Rhodococcus*) are frequently the only bacterial organisms surviving chlorine disinfection of drinking water supplies [102]. Most species of mycobacteria survive exposure to 1 mg/L free chlorine [190,214] and du Moulin et al. [215] found *Mycobacterium marinum* to be resistant to 10 mg/L free chlorine.

Taylor et al. [186] examined the disinfection resistance of five strains of *M. avium* to free chlorine, monochloramine, chlorine dioxide, and ozone (Table 12) and determined the disinfectant concentration multiplied by the contact time (CT) for a 3-log, or 99.9% inactivation (CT_99.9%_). The authors applied centrifugation techniques to minimize mycobacteria clumping that would interfere with disinfection. Free chlorine CT_99.9%_ values for the *M. avium* strains were 700- to 3000-times greater than that for *E. coli*. Similarly, the CT_99.9%_ values of the *M. avium* strains for chlorine dioxide and ozone were at least 100- and 50-fold greater, respectively, compared to the *E. coli* strain. The *M. avium* strains could be divided into two groups based upon their susceptibility to chloramines. Three of the strains were at least six-times more resistant than the *E. coli* C strain, whereas two of the *M. avium* strains were as susceptible to monochloramine as was *E. coli* C.

The results from Taylor et al. [186] demonstrated large variations in sensitivity to disinfection among *M. avium* isolates. Two epidemiology-linked strains (5502 water isolate and 5002 AIDS patient isolate) with the same pulsed-field gel electrophoresis (PFGE) pattern showed markedly different sensitivity patterns to free chlorine, chloramines, and chlorine dioxide. Some of the variability between strains (and various research studies) can be attributed to the degree of clumping, growth conditions, and physiological properties. The authors reported that strain 5502 (water isolate) grew faster than strain 5502 (clinical isolate), and that the slow growth rate could be related to the increased resistance to disinfection. The authors demonstrated that isolates grown in low-nutrient tap water were 4 to 15 times more resistant to free chlorine disinfection than isolates propagated in Middlebrook 7H9 broth. Le Dantec et al. [216] reported that the inactivation rate (k) of *M. gordonae* increased nine-fold when the concentration of the growth medium was diluted ten-fold. The increased disinfection resistance of low nutrient grown organisms has been observed for other waterborne microbes [158].

The US Environmental Protection Agency (USEPA) requires surface water treatment plants to apply filtration and disinfection, primarily as a means of controlling *Giardia* [153,217]. Facilities that use conventional coagulation, sedimentation, and filtration are required to apply sufficient disinfection to achieve at least 0.5 log inactivation of *Giardia* cysts. Data in Table 13 show *M. avium* (average of the five strains in Table 12) to be more resistant than *Giardia* to free chlorine disinfection, but equal or more sensitive than *Giardia* to monochloramine, chlorine dioxide, and ozone. However, both organisms are more sensitive to disinfection than *Cryptosporidium* oocysts [218]. Similar comparisons were made by Jacangelo et al. [219] for 2-logs (99%) inactivation of *M. fortuitum* and *Giardia* (Table 14). The observed level of inactivation for *M. fortuitum* was higher than other reports, in part because the researchers did not attempt to reduce or eliminate bacterial clumps. However, similar to data in Table 13, mycobacteria were 3 to 10 times more resistant than *Giardia* to free chlorine disinfection.

**Figure 16:**
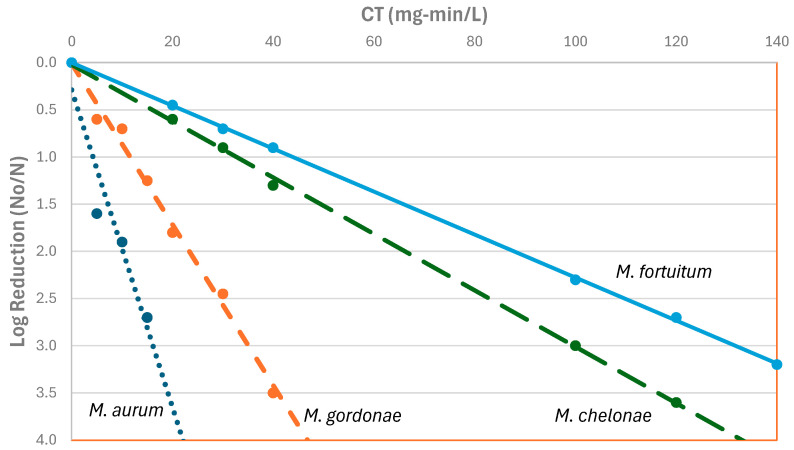
Disinfection of mycobacteria by free chlorine. Experimental conditions: pH 7.0, 25 °C, initial free chlorine concentration 0.5 mg/L.Adapted from Le Dantec et al. [216].

Data for disinfection of other mycobacteria by free chlorine are shown in Table 15. Cells were initially cultured on Middlebrook 7H10 agar but resuspended in sterile reverse osmosis water for 4–5 weeks to adapt the strains to low nutrient conditions. Similar data by Le Dantec et al. [216] are shown in Figure 16. Although the CT values differ between different investigators and replicates of the same species (e.g., *M. chelonae*) showed large variations in disinfection sensitivity, the relative rank of chlorine sensitivity was similar (*M. fortuitum* and *M. chelonae* were more resistant than *M. gordonae*) and, in general, the isolates were more sensitive to free chlorine disinfection than the *M. avium* strains shown in Table 12. Zheng et al. [220] reported that *M. mucogenicum* had CT_99.9_ inactivation values for free chlorine, monochloramine, and chlorine dioxide of 76.3 ± 47.6, 139 ± 382, and 13.5 ± 4.9 mg-min/L, respectively. They attributed the high disinfection resistance to the cell’s high level of hydrophobicity.

Disinfection of a variety of mycobacteria by chloramines was reported by Pelletier et al. [221] (Table 16). The decreasing CT for 0.5-log inactivation with higher chloramine doses suggests that clumping or disinfectant demand may be influencing these results. However, *M. avium* were more resistant than the other mycobacteria examined.

Application of ultraviolet light for microbial inactivation is gaining increased attention, especially for control of *Cryptosporidium*. Inactivation data for mycobacteria by UV light is shown in Table 17. Although sensitivities vary among *Mycobacterium* species, the values are generally in the range for other vegetative bacteria [174]. David et al. [222] reported that *M. tuberculosis* and *M. marinum* were capable of photoreactivation, with an increase of 40 to 56% following irradiation with visible light for 1 h. McCarthy and Schaeffer [223] reported a 1.7-log increase in *M. avium* counts following exposure to 9 mJ/cm^2^ and a 3-h photoreactivation. They also noted that the 14-day post-exposure incubation period for *M. avium* provided ample opportunity for significant dark repair of UV lesions.

Flushing. Hozalski et al. [228] reported that flushing of building plumbing reduced *M. avium* complex (MAC) levels, but that the risks evaluated by QMRA were low (<10^−7^ daily risk threshold). A companion paper by the same research group [229] reported that flushing reduced bacterial levels by 1 to >4 log units, but that bacterial levels largely recovered within a week of post-flush stagnation.

Nutrient control. As already mentioned, environmental mycobacteria are extremely nutritionally diverse and can grow and persist at extremely low nutrient levels. Increases in *M. avium* levels in drinking water distribution systems have been correlated with increased levels of AOC and BDOC [50]. In controlled pilot plant experiments, the effect of biodegradable organic matter on the growth of *M. avium* and natural heterotrophic plate count (HPC) bacteria was investigated [104]. Biofilms of *M. avium* and HPC bacteria formed within 1 week and appeared to stabilize over a 2–3-month period (Table 18). The results showed that the density of biofilm bacteria increased with increasing AOC levels, but that biofilms of *M. avium* readily grew in the absence of a disinfectant residual at AOC levels of 40 µg/L. Therefore, minimizing the level of biodegradable organic matter in drinking water can help limit the density of mycobacteria, but probably cannot prevent their occurrence. Carson et al. [190] reported growth of *M. chelonae* in commercial distilled water to levels of 10^5^ to 10^6^ cells/mL, and levels were maintained over a 1-year period. Collins et al. [230] reported detection of mycobacteria in water softening resins and in water from deionizers. Groundwater typically has low levels of biodegradable organic matter due to the natural removal of nutrients as the water percolates through the soil, however, Covert et al. [211] reported detecting *M. gordonae* in 31% of 16 groundwater systems. Falkinham et al. [50] detected *M. avium* and *M. intracellulare* in 6% and 3% of the samples from a groundwater system with average AOC levels of 28 µg/L and an average 0.09 mg/L free chlorine residual at the end of the system [213]. However, King et al. [192] did not detect *M. avium* or *M. intracellulare* in treated groundwater samples.

**Figure 17:**
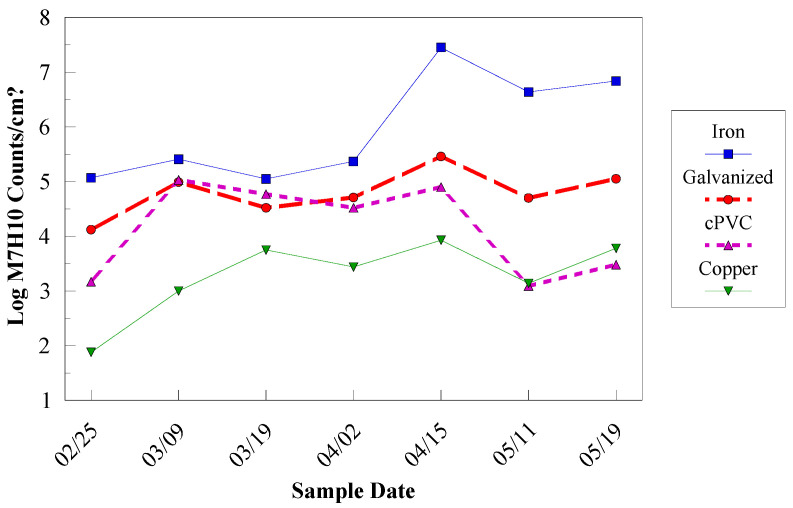
Impact of pipe composition on biofilm growth of *M. avium*. Adapted from LeChevallier et al. [231].

Pipe Materials/Corrosion Control. The impact of pipe composition on the biofilm growth of *M. avium* is shown in Figure 17. *M. avium* levels were higher on iron and galvanized pipe than on PVC or copper surfaces. The resistance of *M. avium* to zinc may have enhanced its survival on galvanized pipe surfaces [103].

The effect of the combination of biodegradable organic matter, disinfectant type, and pipe composition on the survival of *M. avium* and HPC bacteria is shown in Table 3. The lowest level of biofilms occurred on copper under low nutrient and low free chlorine conditions. Chloramines controlled biofilms on iron pipes better than free chlorine, particularly under low-nutrient conditions. As noted above, several researchers have suggested that use of chloramines as a post disinfectant may select for the presence of *M. avium* in drinking water supplies, although many systems that convert to chloramines do so because of high levels of organic carbon in the water supply that form chlorinated disinfection by-products.

Tolofari et al. [204] compared the disability-adjusted life year (DALY) risk of a *M. avium* infection to the risk from disinfectant by-products (total trihalomethanes and total haloacetic acids) and found that the potential health risks from disinfection byproducts for bladder cancer (an annual DALY of 7.61 × 10^−7^) was five orders of magnitude greater than the annual DALY of MAC pulmonary disease (4.74 × 10^−12^). The authors noted that while the results were specific to the system considered (e.g., chloramine disinfectant, no detectable *Legionella*), the study presents a framework for prioritizing microbial and chemical risks.

Association with Free Living Amoebae. Like *Legionella*, environmental mycobacteria are known to infect free-living amoebae [232,233] where such growth can increase its virulence and provide protection from antimicrobial agents [234,235]. Protozoan intracellular growth rates of *M. avium*, *M. intracellulare*, and *M. scrofulaceum* were 4 to 40-fold faster than compared to water-grown isolates [236]. Therefore, it is possible that efforts to control the growth of free-living amoeba and protozoa will have beneficial effects for reducing the risk of mycobacteria in drinking water. Few studies have specifically focused on the control of free-living protozoa or the effect of control measures on *Mycobacterium* risk, although it is likely that efforts to reduce biofilm levels in drinking water pipelines would also result in lower amoeba and protozoa levels because these organisms feed on biofilm bacteria. Additional studies are needed in this area.

### 5.2. Pseudomonas aeruginosa

Description. *P. aeruginosa* is a non-fermentative, gram-negative bacterium commonly found in untreated water and soils and can be regularly found on the surfaces of plants and some animals. The bacterium almost never infects a healthy person but is most commonly associated with infections in patients with cystic fibrosis, burn wounds, immunodeficiency, chronic obstructive pulmonary disorder (COPD), cancer, and severe infection requiring ventilation, such as COVID-19. *P. aeruginosa* can cause infections in the blood, lungs (pneumonia), or other parts of the body after surgery. Its significance as a pathogen is exacerbated by its resistance to antibiotics, virulence factors, and its ability to adapt to a wide range of environments. *P. aeruginosa* is nutritionally diverse and can grow in distilled water and is resistant to a wide variety of environmental stressors, including causing an outbreak by growth in a concentrated antiseptic [237,238]. *P. aeruginosa* is one of the top-listed pathogens causing hospital-acquired infections, which are widely attributed to medical devices (ventilation) because they tend to thrive on wet surfaces [239]. Antibiotic-resistant *P. aeruginosa* is listed among the “critical” group of pathogens by the World Health Organization (WHO).

In 2017, multidrug-resistant *P. aeruginosa* caused an estimated 32,600 infections among hospitalized patients and 2700 estimated deaths in the United States [240]. Patients with *P. aeruginosa* infections had significantly longer hospitalizations, higher mortality, and overall higher cost of treatment compared to patients without an infection [241].

The environmental survival of *P. aeruginosa* is enhanced by the production of an extracellular polysaccharide alginate that protects the cells from disinfection, particularly when grown as biofilms on a pipe surface [242]. In fact, chlorine exposure of *P. aeruginosa* stimulates certain genes in response to the stressor that increases the resistance of the organism to antibiotics [243]. Although *P. aeruginosa* can be found in potable water, sinks and drains, and vegetation (e.g., flowers and uncooked vegetables) in healthcare settings, studies show that the organism is typically transmitted on the hands of hospital staff, illustrating the need to adhere to a strict hand disinfection policy [244]. Although contamination of recreational waters and tap water has been associated with outbreaks of *P. aeruginosa*, the relative role water plays in the transmission of this bacterium to humans is still unclear. *P. aeruginosa* is not regulated for municipal drinking water because there is no evidence that it is a source of infection for the general population [14]. France has recommended levels be less than 1 cfu/100 mL for water used in healthcare facilities [245]. Croatia, Serbia, and Montenegro are countries that have *P. aeruginosa* included in their national regulations for drinking water and bottled water [246].

**Figure 18:**
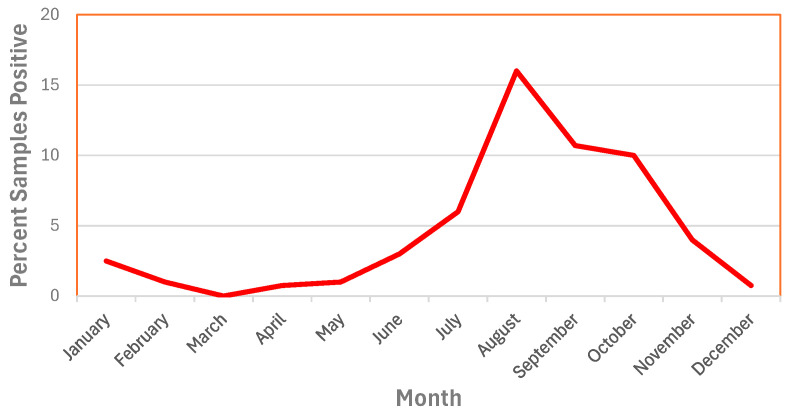
Seasonal occurrence of P. aeruginosa in Croatia. Adapted from Vukić Lušić et al. [246].

Occurrence in Distribution Systems. Wang et al. [210] detected *P. aeruginosa* by qPCR in 3 of 144 samples collected from houses in two chloraminated systems, and levels were reduced 10-fold when the sample taps were flushed, such that only 1 sample was positive after flushing the tap for 3 min. The authors concluded that *P. aeruginosa* occurrence was rare in drinking water distribution systems. Lu et al. [87] detected *P. aeruginosa* by qPCR in 22% (4 of 18) of sediment samples collected from water storage tanks in 10 states in the U.S. Genomic levels averaged 250 cell equivalents per gram. By comparison, *L. pneumophila* was detected far more frequently (33%). Vukić Lušić et al. [246] analyzed 4171 drinking water samples for *P. aeruginosa* by culture (ISO 16266:2008) and detected the organism in 3.9% of the samples, primarily during building renovation or new construction and the commissioning of a new water supply network. The median levels of *P. aeruginosa* were 9 cfu/100 mL in the new supply network and 4 cfu/100 mL in the buildings. *P. aeruginosa* occurrence was highest during the late summer and early fall (Figure 18). A negative correlation was found between *P. aeruginosa* and free chlorine, but 98% of the positive samples had residual chlorine concentrations within the range of 0.2–0.25 mg/L [246].

Anversa et al. [247] cultured *P. aeruginosa* on m-PA-C agar from three distribution systems in Brazil and detected the organism in 7.6% (19/251) of the water samples. All the positive *P. aeruginosa* occurred with chlorine concentrations between 0.2 and 2.0 mg/L, suggesting resistance to the disinfectant. The authors showed a significant relationship between *P. aeruginosa* and total coliform bacteria, with nearly half of the *P. aeruginosa* positive samples also showing detection of total coliform bacteria (there was no correlation to the presence of *E. coli*).

Papapetropoulou et al. [248] recovered *P. aeruginosa* in 9% (8/88) drinking water samples from water systems in Greece and reported that the strains were resistant to streptomycin and tetracycline, and were frequently accompanied by resistance to chloramphenicol, erythromycin, and nalidixic acid.

Risk Management. Risk models for *P. aeruginosa* are not well developed because it is likely that a different model is needed for each route of exposure. *P. aeruginosa* may enter the body though almost any ‘exposed’ tissue, including the skin, ears, eyes, urinary tract, lungs, and the gut [249]. The oral infectious dose for *P. aeruginosa* in drinking water has been estimated to be in the range of 10^8^ to 10^9^ cfu [203]. George et al. [250] found that the aerosolized intranasal LD_50_ dose for mice was 2.7 × 10^7^ cfu. In the case of pool associated folliculitis outbreaks, the hazardous levels for healthy individuals have been suggested to be greater than 10^3^ to 10^6^ cfu/mL [251]. Therefore, the goal for risk management for *P. aeruginosa* in drinking water should be to limit the occurrence of the organism to low levels far below these risk thresholds.

Disinfection. Bédard et al. [252] conducted an excellent review of *P. aeruginosa* in large buildings, and the paper concisely summarized the literature for various disinfection control strategies (Table 19). Although resistance to free chlorine will vary depending on the strain, biofilm associated *P. aeruginosa* will survive chlorination at concentrations that are applicable to drinking water. As previously mentioned, extracellular polymeric substances (EPS) increase the resistance of *P. aeruginosa* cells, particularly in biofilms. Disinfectants need to penetrate the biofilm material before they can interact with the cell membrane to inactivate the bacterium. Monochloramine is considered more effective against biofilms of *P. aeruginosa*; a chloramine dose of 4 mg/L with 1 h. of contact time resulted in a 4-log reduction compared to a 2-log reduction with 5.8 mg/L of free chlorine for the same contact time (Table 19). A study on multispecies biofilms from drinking water demonstrated the high level of resistance of *P. aeruginosa*, requiring up to 600 mg Cl_2_/L to reduce their occurrence to below detectable levels [253]. Chlorine dioxide and ozone are also effective disinfectants for *P. aeruginosa* control (Table 19). The Australian Drinking Water Guidelines recommend maintenance of a free chlorine residual of at least 0.2 mg/L in the distribution system to control *P. aeruginosa* [1].

Nutrient Control. The metabolic diversity of *P. aeruginosa* was illustrated by the study by van der Kooij et al. [254], which reported that 5 strains could use 31 of the 35 low-molecular weight compounds (including amino acids, carbohydrates, carboxylic acids, and aromatic acids) tested at levels of 25 µg/L. When inoculated into various tap waters, two of the *P. aeruginosa* strains were able to grow to levels ranging from 10^2^ to 10^5^ cfu/mL. van der Wielen and van der Kooij [255] detected low levels of *P. aeruginosa* in undisinfected distribution systems in The Netherlands that used two surface water and had AOC levels > 20 µg/L. Groundwater, which typically has low levels of AOC and BDOC, typically had no or low levels of *P. aeruginosa* occurrence. Allen and Geldreich [256] detected *P. aeruginosa* in only 3% of 700 samples from drinking water systems, mostly from groundwater sources. Wang et al. [210] reported low occurrence of *P. aeruginosa* by qPCR in a chloraminated distribution system that was a blend of surface, ground, and desalinated water. *P. aeruginosa* was not detected by culture in biofilms that were sampled over an 18-month study from 18 pipes made of various materials in systems distributing non-chlorinated groundwater [257].

Flushing/cleaning. The Water Services Association of Australia [258] recommends good management practices such as maintaining disinfectant residuals, flushing and disinfection after repairs, and regular cleaning of sediment from pipes and storage tanks to reduce the opportunities for regrowth of *P. aeruginosa* in distribution systems. Wang et al. [210] reported a 10-fold reduction in *P. aeruginosa* by qPCR when sample taps were flushed. Van Bel et al. [259] reported that flushing alone without additional chlorination was not sufficient to remove microorganisms from a pilot distribution system.

#### Other Opportunistic Pathogens

Brief consideration is given to the following opportunistic pathogens that can also be associated with drinking water. The public health risk for these microbes in drinking water is still unclear, but it is prudent to manage drinking water systems to also limit these risks in addition to the microbes listed above. In most cases, efforts to manage *Legionella*, mycobacteria, and *Pseudomonas* risks will also be effective for the following microorganisms.

*Aeromonas hydrophila. Aeromonas* is a genus of Gram-negative, facultative anaerobic, rod-shaped bacteria that morphologically resemble members of the family *Enterobacteriaceae. Aeromonas* species can frequently be recovered in some of the lactose-fermenting total coliform assays. At least 36 species have been described in the genus *Aeromonas*, of which at least 19 are considered emerging pathogens for humans [260]. The most important pathogens are *A. hydrophila*, *A. caviae*, *A. dhakensis*, and *A. sobria*. Aeromonads occur naturally in a wide variety of environments including fresh and salt water, treated and raw sewage water, plumbing systems, fish, shellfish, domestic animals, raw meat, and vegetables. Major diseases associated with *Aeromonas* are gastroenteritis and wound infections. The most vulnerable people to an *Aeromonas* infection include those with weak immune systems or other underlying illnesses, young children, and the elderly. Potential virulence factors (e.g., endotoxins, hemolysins, enterotoxins, adherence factors) have been identified with clinical strains of *Aeromonas* [261].

*Aeromonas* spp. are ubiquitous in rivers and freshwater lakes and have frequently been observed in drinking water systems [262]. *Aeromonas* isolates have been reported to be more susceptible to chlorine disinfection than for *E. coli*. *Aeromonas* was widely detected in non-chlorinated drinking water in the Netherlands, but studies with phenotyping and genotyping methods showed that the environmental isolates were different from clinical isolates [263]. The authors reported that *Aeromonas* isolates were mainly associated with sediments and loose deposits in the distribution system, so that flushing would be effective for *Aeromonas* management. The Environmental Protection Agency (EPA) in the United States removed *Aeromonas* from the contaminant candidate list (CCL3) in 2009 [264]. The presence of *Aeromonas* in drinking water is currently not considered a health-related problem [14]. The Australian Drinking Water Guidelines recommends maintenance of a free chlorine residual of at least 0.2 mg/L in the distribution system to control *Aeromonas* [1].

*Klebsiella pneumoniae*. *Klebsiella* is a genus of Gram-negative, oxidase-negative, rod-shaped bacteria with a prominent polysaccharide-based capsule that promotes resistance to disinfection [158]. Habitats include wastewater, drinking water, soil, surface waters, industrial discharges, and vegetation. In addition, *Klebsiella* species can be routinely found in the human nose, mouth, and gastrointestinal tract as normal flora [265]. *Klebsiella* organisms can lead to a wide range of disease states, including pneumonia, urinary tract infections, sepsis, meningitis, diarrhea, peritonitis, and soft tissue infections [266]. The majority of human *Klebsiella* infections are caused by *K. pneumoniae* and *K. oxytoca*. Infections are more common in the very young, very old, and those with other underlying diseases, such as cancer, and most infections involve contamination of an invasive medical device [267]. *Klebsiella* infections can be a concern due to the increasing incidence of antibiotic resistance.

*Klebsiella* is one of the four common genera included in the total coliform population and can be commonly recovered from drinking water supplies. Mackay [268] reported *Klebsiella* in more than 75% of water samples in the Sydney water system. The survival of the organism was enhanced by association with sediments and deposits in water mains. LeChevallier et al. [158] reported that the attachment of unencapsulated *K. pneumoniae* grown in a high nutrient medium to glass microscope slides afforded the microorganisms as much as a 150-fold increase in disinfection resistance and that disinfection resistance was also affected by the age of the biofilm, bacterial encapsulation, and previous growth conditions (e.g., growth medium and growth temperature). These factors were found to increase resistance to chlorine by 2- to 10-fold. Goel and Bouwer [269] reported similar results and that the presence of manganese and iron in the growth medium resulted in the highest resistance to free chlorine with CT_99.9_ values of 13.1 and 13.8 mg-min/L, respectively.

*Klebsiella* spp. are not considered to represent a source of gastrointestinal illness in the general population through ingestion of drinking water. *Klebsiella* spp. detected in drinking-water are generally biofilm organisms and are unlikely to represent a health risk [14]. Growth in distribution systems can be minimized by strategies designed to minimize biofilm growth, including treatment to optimize organic carbon removal, restriction of the residence time of water in distribution systems, flushing and cleaning of water mains and storage tanks, and maintenance of a disinfectant residual.

*Serratia marcescens. S. marcescens* is a species of rod-shaped, gram-negative bacteria in the family *Yersiniaceae*. *S. marcescens* occurs naturally in soil, water, air, plants, and animals, and produces a red pigment (prodigiosin) at room temperature. It is commonly involved in hospital-acquired infections, particularly catheter-associated bacteremia, urinary tract infections, and wound infections [270]. Most clinical strains were resistant to several antibiotics. Historically, *S. marcescens* was considered non-pathogenic and in the 1950s, the US Army filled balloons with *S. marcescens* and burst them over San Francisco to study wind patterns that might affect biological warfare. Shortly afterward, doctors noted a dramatic increase in pneumonia and urinary tract infections [271].

*Serratia* sp. are commonly recovered as part of the total coliform group [272,273] and can be routinely detected in drinking water supplies. Sometimes, the red pigmented film in toilet bowls or shower curtains are attributed to *S. marcescens* [274]. Procedures for managing total coliform bacteria in distribution systems (i.e., maintenance of a disinfectant residual, flushing and cleaning of tanks, etc.) should be adequate for controlling *S. marcescens* levels.

*Burkholderia pseudomallei. B. pseudomallei* (also known as *P. pseudomallei*) is a gram-negative, bipolar, aerobic, motile rod-shaped bacterium. It is a soil-dwelling bacterium endemic in tropical and subtropical regions worldwide, particularly in Thailand and northern Australia [41]. Infections, called melioidosis, occur most commonly during the wet season in individuals with diabetes, liver disease (due to alcohol use), kidney disease, cancer, or chronic lung disease (such as cystic fibrosis, COPD, or bronchiectasis). The bacterium commonly infects the lungs (pneumonia), but the infection may involve almost any organ, with the skin and soft tissues, genitourinary system, visceral organs, and bone and joints typically affected [41]. In Australia, the incidence of melioidosis varies by region, and the case rates range between 5.4 and 50.2/100,000 population [41].

The organism was shown to survive in distilled water for 16 years, demonstrating that it is capable of prolonged persistence in water and resistant to a variety of harsh conditions, including nutrient deficiency, extreme temperature, or pH [275]. Outbreaks of melioidosis in Australia after exposure to contaminated water have been described, mostly in unchlorinated water [276] or in systems where chlorination was not properly maintained. Mayo et al. [277] sampled 55 unchlorinated bore waters in northern Australia and found that 18 (33%) were culture positive for *B. pseudomallei*. Howard and Inglis [278] reported that *B. pseudomallei* was inactivated by 4 logs with a 1 mg/L free chlorine dose in 30 min. Co-culture of *B. pseudomallei* with *Acanthamoeba astronyxis* provided a 100-fold increase in chlorine resistance. O’Connell et al. [279] examined eight environmental and clinical isolates of *B. pseudomallei* in phosphate-buffered water and reported the highest CT values for a 4-log_10_ inactivation were 7.8 mg-min/L for free chlorine at pH 8 and 5 °C and 550 mg·min/L for monochloramine at pH 8 and 5 °C.

*Acinetobacter baumannii. A. baumannii* is a gram-negative bacillus that is aerobic, pleomorphic, and non-motile. *A. baumannii* has a high incidence among immunocompromised individuals, particularly those who have experienced a prolonged (>90 d) hospital stay. The organism has been shown to colonize the skin as well as being isolated in high numbers from the respiratory and oropharynx secretions of infected individuals [280]. Concern has increased with the increased levels of antibiotic resistance. According to the CDC, carbapenem-resistant *A. baumannii* cause an estimated 8500 infections in hospitalized patients and 700 estimated deaths in the United States [281]. Although *Acinetobacter* species are routinely detected in food, water, and soil, the transmission of *A. baumannii* from drinking water is less clear [282]. *A. baumannii* has been isolated from untreated surface water [283,284], wastewater [284,285], and from sinks (but not the hot or cold-water plumbing) of an intensive care unit of a hospital in Tokyo, Japan [286], and a showerhead in Mexico City, Mexico [287]. Maki et al. [288] detected *A. baumannii* in several point-of-use filters in Flint, Michigan; however, their effect on bacterial infections were not studied.

Bench-scale studies of the inactivation of *A. baumannii* showed that free chlorine and monochloramine could achieve 3-log_10_ with the CT values of 10 mg-min/L, with free chlorine more effective than monochloramine [289]. Karumathil et al. [290] reported complete survival of *A. baumannii* to a dose of 4 mg/L of free chlorine for 120 s, but the use of very high cell concentrations (10^9^ cells) likely created a large chlorine demand (chlorine residuals were not measured after exposure).

*Stenotrophomonas maltophilia. S. maltophilia* is an aerobic, nonfermentative, gram-negative bacterium. Initially classified as *Bacterium bookeri*, then renamed *Pseudomonas maltophilia*, it was grouped in the genus *Xanthomonas* before eventually becoming the type species of the genus *Stenotrophomonas* [291]. *S. maltophilia* is frequently isolated in the environment, particularly from water bodies like rivers, wells, and lakes, as well as bottled water, sewage, swine/chicken feces, soil, plants, salads, frozen fish, and raw milk [292]. It frequently colonizes humid surfaces such as the tubes used in mechanical ventilation, indwelling urinary catheters, and medical devices such as suction catheters and endoscopes. It can be found in multiple healthcare settings, such as hospital tap water faucets, sinks, shower outlets, air-cooling systems, and ice-making and soda fountain machines [291]. *S. maltophilia*’s significance as a nosocomial pathogen comes from its resistance to antibiotics. A ten-year review of *S. maltophilia* cases in a Melbourne Australia hospital showed that 80% of the infections were acquired while the patient was in the hospital (nosocomial), suggesting that community-acquisition was possible [293]. *S. maltophilia* is estimated to cause about 1% of nosocomial bacteremia cases and incidence of infection is estimated to be 5.7 to 37.7 cases per 10 thousand hospital discharge patients [291].

Risk factors for *S. maltophilia* infection include chronic respiratory diseases (COPD), cystic fibrosis, cancer or organ transplant patients, human immunodeficiency virus (HIV) infection, hemodialysis patients, and newborns. Prolonged stays in intensive care, mechanical ventilation, tracheostomies, central venous catheters, severe traumatic injuries, significant burns, mucosal barrier damaging factors, and the use of broad-spectrum antibiotics increased the risk of infection [291].

van der Wielen and van der Kooij [255] detected *S. maltophilia* by qPCR in five of eight unchlorinated distribution systems examined at concentrations ranging from below detection (<400) to 1 × 10^4^ gene copies/L. Similarly, *S. maltophilia* was detected in three of five sources of bottled spring water in France [294]. *S. maltophilia* was the most identified member of the heterotrophic plate count population recovered from showerheads in one South Korean city [295]. Free chlorine residuals were typically <0.3 mg/L. The growth and survival of *S. maltophilia* was enhanced by association with free-living amoeba (*Vermamoeba vermiformis*) and the bacterium was recovered from amoeba-derived structures after 28 days [296].

*Arcobacter butzleri*. The species *A. butzleri*, previously named *Campylobacter butzleri*, can be found in environmental samples; untreated water appears to be a potential source of infection [297]. In industrialized countries, however, the most important source of human contamination may be food. *A. butzleri* has been isolated in different breeding animals and is present in a great variety of retail meats, including chicken, beef, pork, and lamb, with a high prevalence in poultry [298]. In humans, *A. butzleri* has been linked to gastrointestinal infections and occasionally infections of the blood. Symptoms include diarrhea associated with abdominal pain, nausea, and vomiting or fever. Without treatment, patients infected with *A. butzleri* can experience symptoms for two days to several weeks, but with antimicrobial therapies the infection can be eradicated within a few days [299].

For three reported waterborne outbreaks associated with *Arcobacter* that occurred in Finland, Slovenia, and the US, the bacterium was isolated either from the drinking water or the feces of patients with diarrhea [297]. In all cases, the drinking water was fecally contaminated. *Arcobacter* sp. has been detected in various water samples, such as groundwater, river water, wastewater, canal water, seawater, spring water, and drinking water, with the highest detection in sewage. *Arcobacter* detection rates are nearly 100% in wastewater samples in tested countries, such as Italy, Spain, the UK, and the US [297,299]. Failures in the chlorination process for drinking water have been considered the cause of at least one outbreak and removal of *Arcobacter* by traditional drinking water treatment/ disinfection has been shown to be highly effective [300]. *Arcobacter* is susceptible to chlorination with at least 5 log_10_ reduction in culturability after a 5-min exposure [301,302].

Free-Living Amoeba. Although not well studied, free-living amoeba (FLA) are common in drinking water supplies. Thomas and Ashbolt [232] published an excellent review that showed FLA could break through (or by-pass via intrusion) water treatment barriers and enter distribution systems where there is colonization and regrowth, especially in reservoirs and in-premise plumbing storage tanks. Depending on the analytical methodology and the type of water system, the average FLA detection rate was 45% at the customer tap. Nearly all the opportunistic pathogens covered in this review have been reported to associate or grow within FLA. This interaction is thought to be linked to the increased virulence for these bacteria [232].

In addition to being a host for these microbes, the FLA may also have public health risks as opportunistic pathogens themselves. *Naegleria fowleri*, commonly known as the “brain-eating amoeba”, is a free-living amoeba found in warm freshwater and soil (30–45 °C). *N. fowleri* can cause a rare, devastating infection of the brain called primary amebic meningoencephalitis (PAM) caused by water entering the nose and the amoeba traveling to the brain. The risk of an *N. fowleri* infection is greater when one swims in polluted freshwater lakes or streams. Several cases have also been found when inadequately disinfected tap water has entered the nose, such as when one rinses the sinuses using a neti pot, or when cleansing the nose with water for religious practices [303]. It is recommended that water used for any home medical procedures first be boiled then cooled.

The Australian Drinking Water Guidelines recommends that water supplies at risk of *N. fowleri* should provide adequate primary disinfection (a CT > 30 mg-min/L) and maintain a chlorine residual of at least 0.5 mg/L throughout the distribution system to control *N. fowleri* [1]. The guidelines suggest that a density of 2 *N. fowleri* organisms per liter is an appropriate threshold for action in drinking water supplies. *N. fowleri* is a concern in Northern Australia where temperatures can exceed 30 °C. Bartrand et al. [304] provides an excellent review of *N. fowleri* control in treatment and distribution systems.

One of the most common amoebae, *Acanthamoeba*, is a microscopic, free-living amoeba found naturally in dust and soil, fresh and salt-water, as well as building plumbing, heating, air-conditioning, and humidifier systems. Although rare, an infection of *Acanthamoeba* can become severe, infecting the eye (Acanthamoeba keratitis), brain, and spinal cord (Granulomatous encephalitis), and can spread throughout the entire body. Acanthamoeba keratitis (AK) is an eye infection caused from poor hygiene practices of contact lens wearers and can potentially lead to blindness caused by infection of the eye. Contact lens wearers should never rinse the lens or cases with tap water. Facility managers who oversee environments where eye wash stations are required need to include those stations in their regular cleaning and maintenance program and follow any guidelines offered for eye wash stations. *Acanthamoeba* can form dormant cysts, which are highly resistant to disinfections and temperature. Additional guidance is provided by the USEPA [305] and the CDC [306].

*Acanthamoeba* cysts are extremely resistant to chlorine. De Jonckheere and Van de Voorde [147] reported that it required 16 mg/liter within one hour to achieve a 99.99% (4 log_10_) inactivation of *Acanthamoeba* cysts. However, a dose of 1.0 mg/L free chlorine with a residual of 0.25 mg/liter after 30 min resulted in a 99.99% reduction of *A. castellanii* trophozoites at pH 7.0 and 25 °C [307]. Additional data on disinfection of various amoeba are found in Table 6, Table 7, Table 8, Table 9 and Table 10 for free chlorine, chloramines, chlorine dioxide, ozone, and UV.

## 6. Conclusions and Recommendations

This report provided a review of the three most common opportunistic pathogens in drinking water systems (*L. pneumophila*, *M. avium*, and *P. aeruginosa*) and additional information on a group of nine other less common opportunistic pathogens (*A. hydrophila*, *K. pneumoniae*, *S. marcescens*, *B. pseudomallei*, *A. baumannii*, *S. maltophilia*, *A. butzleri*, *N. fowleri*, and *Acanthamoeba*). The report found that the characteristics of opportunistic pathogens that make them problematic for water treatment include: (1) they are normally present in aquatic environments, (2) they grow in biofilms that protect the bacteria from disinfectants, and (3) under appropriate conditions in drinking water systems (e.g., warm water, stagnation, low disinfectant levels, etc.) these bacteria can amplify to levels that can pose a public health risk.

The approach to managing opportunistic pathogens in drinking water supplies focuses on controlling the growth of these organisms. Many of these microbes are normal inhabitants in biofilms in water, so the attention is less on eliminating these organisms from the system and more on managing their occurrence and concentrations. With anticipated warming trends associated with climate change, the factors that drive the growth of opportunistic pathogens in drinking water systems will likely increase. It is important, therefore, to evaluate treatment barriers and management activities for control of opportunistic pathogen risks. Controls for primary treatment, particularly for turbidity management and disinfection, should be reviewed to ensure adequacy for opportunistic pathogen control.

However, the major focus for a utility’s opportunistic pathogen risk reduction plan should be the management of biological activity and biofilms in the distribution system. Factors that influence the growth of microbes (primarily in biofilms) in the distribution system include, temperature, disinfectant type and concentration, nutrient levels (measured as AOC or BDOC), stagnation, flushing of pipes and cleaning of storage tank sediments, and corrosion control. Pressure management and distribution system integrity are also important to the microbial quality of water but are related more to the intrusion of contaminants into the distribution system, rather than directly related to microbial growth.

Table 20 summarizes the key elements presented in this review for each of the opportunistic pathogens addressed. These key elements include the identified risk from drinking water, the availability and quality of disinfection data for treatment, and guidelines or standards for control. For *L. pneumophila* the risk for this organism has been clearly established from drinking water, cases have increased worldwide, and it is one of the most identified causes of drinking water outbreaks. Water management best practices (e.g., maintenance of a disinfectant residual throughout the distribution system, flushing and cleaning of sediments in pipelines and storage tanks, among others) that have been shown to be effective for control of *L. pneumophila* in water supplies. In addition, there are well documented management guidelines available for the control of the organism in drinking water distribution systems.

By comparison, management of risks for *Mycobacteria* from water are less clear than for *L. pneumophila*. Treatment of *M. avium* is difficult due to its resistance to disinfection, the tendency to form clumps and attachment to surfaces in biofilms. Additionally, there are no guidelines for management of *M. avium* in drinking water and one risk assessment study suggested a low risk of infection (Tolofari et al., 2022) [204]. The role of tap water in the transmission of the other opportunistic pathogens reviewed in this report is less clear and in many cases actions to manage *L. pneumophila* (e.g., maintenance of a disinfectant residual, flushing, cleaning of storage tanks, etc.) will also be beneficial in helping to manage these organisms as well. Clearly, additional research is desperately needed to fill these important public health gaps.

## Figures and Tables

**Table 1:** Relationship Between Threshold Criteria and Growth of Coliform Bacteria in Distribution Systems.

Number of Positive Criteria	Total Number of Events	Coliform Positive Samples	Number of Coliform Episodes	Frequency of Coliform Observation (%)
0	160	3	3	1.9
1	292	18	15	5
2	191	24	16	8.4
3	62	26	10	16

From Volk and LeChevallier [29]. Data based on 95 water utilities.

**Table 2:** Physicochemical data before and after flushing of locations in an Australian distribution system.

Site	Lancelot Crt 12		Glenview Crt 7		Windmill Rise		Warrensbrook Rd	
Flush	Before	After	Before	After	Before	After	Before	After
Date	3 November 2021	3 November 2021	4 November 2021	4 November 2021	9 November 2021	9 November 2021	10 November 2021	10 November 2021
Cl_2_ total	0.76	0.71	0.68	0.73	NA	NA	NA	NA
Cl_2_ free	0.62	0.63	0.60	0.65	NA	NA	NA	NA
Turbidity NTU	255.00	1.62	2.16	1.26	22.00	1.30	NA	NA
ATP *	48.00	1.60	2.37	1.35	27.00	1.40	19.00	0.93
HPC 22C **	160.00	100.00	NA	NA	320.00	1200.00	610.00	0.00
HPC 37C	61.00	59.00	NA	NA	33.00	63.00	17.00	0.00
W-COD mg/L	53.00	2.00	NA	NA	10.00	2.00	25.00	2.00
Alkalinity mg CaCO_3_/L	18.00	14.00	NA	NA	14.00	14.00	45.00	15.00
TKN mg N/L	0.20	0.10	NA	NA	0.40	0.00	2.10	0.00
TP mg P/L	0.11	0.08	NA	NA	0.13	0.00	0.56	0.10
Fe	0.00	0.11	NA	NA	2.90	0.13	21.00	0.14
MN	0.00	0.00	NA	NA	0.33	0.01	1.40	0.01
Ti	0.00	0.01	NA	NA	0.06	0.00	0.33	0.00
Zn	0.00	0.00	NA	NA	0.03	0.00	0.36	0.00
AL	0.00	0.16	NA	NA	1.50	0.09	9.90	0.10
Ba	0.00	0.02	NA	NA	0.04	0.02	0.18	0.02
Ca	4.80	4.50	NA	NA	5.80	5.60	8.30	5.10
K	0.80	0.60	NA	NA	0.90	0.70	1.90	0.70
Mg	1.40	1.10	NA	NA	1.60	1.40	3.10	1.30
Cu	0.00	0.00	NA	NA	0.00	0.00	0.03	0.00
AS	0.00	0.00	NA	NA	0.00	0.00	0.01	0.00
Co	0.00	0.00	NA	NA	0.00	0.00	0.02	0.00
Cr	0.00	0.00	NA	NA	0.00	0.00	0.02	0.00
Ni	0.00	0.00	NA	NA	0.01	0.00	0.04	0.00
Sr	0.00	0.02	NA	NA	0.02	0.02	0.04	0.02
V	0.00	0.00	NA	NA	0.00	0.00	0.03	0.00
Pb	0.00	0.00	NA	NA	0.00	0.00	0.01	0.00
B	0.01	0.05	NA	NA	0.06	0.06	0.05	0.07
Zn	0.00	0.00	NA	NA	0.00	0.00	0.01	0.00
Na	5.10	5.20	NA	NA	6.20	6.20	6.30	6.00
pH	7.40	7.60	NA	NA	7.50	7.60	NA	NA

All metals are in mg/L. NA no data available. * ATP unit not provided; it is assumed this relates to Adenosine Triphosphate as a proxy for microbial activity. ** Heterotrophic Plate Count at 22 °C.

**Table 3:** Impact of nutrient level, disinfectant, and pipe material on *M. avium* and HPC levels.

AOC Level	Disinfectant Type	Disinfectant Residual (mg/L)	Copper Pipe ^1^		Iron Pipe ^1^	
			HPC	*M. avium*	HPC	*M. avium*
85 μg/L AOC	Free chlorine	0.6	1.76	0.18	6.02	5.85
	Chloramine	2.2	2.44	2.38	5.21	4.92
213 μg/L AOC	Free chlorine	0.3	2.17	0.37	5.93	5.50 *
	Chloramine	1.4	2.43	2.10	5.89	5.20 *

^1^ Values are log cfu/cm^2^, * Corrosion products interfered with these analyses. Adapted from Norton et al. [104].

**Table 4:** ECDC Legionnaires’ disease in Europe, Surveillance Report 2020.

Species	Number in 2020	Percent of Cases
*L. pneumphila*	843	95.2
*L. anisa*	1	0.1
*L. bozemanii*	6	0.7
*L. longbeachae*	17	1.9
*L. micdadei*	7	0.8
L. other species	11	1.2
L. species unknown	15	1.7

Source: https://www.ecdc.europa.eu/en/legionnaires-disease (accessed on 1 May 2023).

**Table 5:** CDC Guidance on Legionella Occurrence in Potable Water. From CDC [18].

Concentration indicates that *Legionella* growth appears:					
Uncontrolled	Poorly Controlled	Well Controlled			
≥10 CFU/mL in potable water	1.0-9.9 CFU/mL in potable water	Detectable to 0.9 CFU/mL in potable water	No *Legionella* detected in a single round of testing	No *Legionella* detected in multiple rounds of testing	No *Legionella* detected in multiple rounds of testing with methods that detect viable and non-viable bacteria of any Legionella species
**Change in concentration over time indicates that *Legionella* growth appears:**					
**Uncontrolled**	**Poorly Controlled**	**Well Controlled**			
100-fold or greater increase in concentration	10-fold increase in concentration	*Legionella* concentration steady for two consecutive sampling rounds	No *Legionella* detected in a single round of testing	No *Legionella* detected in multiple rounds of testing	No *Legionella* detected in multiple rounds of testing with methods that detect viable and non-viable bacteria of any Legionella species
**Extent indicated that *Legionella* growth appears:**					
**Uncontrolled**	**Poorly Controlled**	**Well Controlled**			
Detection in multiple locations AND a common source location OR Detection across many locations within a water system	Detection in a common source location that serves multiple areas OR Detection in more than one location within a water system	Detection in a few of many locations within a water system	No *Legionella* detected in a single round of testing	No *Legionella* detected in multiple rounds of testing	No *Legionella* detected in multiple rounds of testing with methods that detect viable and non-viable bacteria of any Legionella species

**Table 6:** Chlorine Inactivation of *L. pneumophila* and Acanthamoeba Trophozoites.

Organism	Temperature (°C)	pH	CT_99.9%_
*Acanthamoeba* M3	30	8	12
*Acanthamoeba* M3 (infected)	30	8	5
*Acanthamoeba* S2	30	8	37
*Acanthamoeba* S2 (infected)	30	8	39
*Acanthamoeba* V1	30	8	70
*Acanthamoeba* V1 (infected)	30	8	82
*Acanthamoeba* M3	50	8	5
*Acanthamoeba* M3 (infected)	50	8	5
*Acanthamoeba* S2	50	8	5
*Acanthamoeba* S2 (infected)	50	8	5
*Acanthamoeba* V1	50	8	28
*Acanthamoeba* V1 (infected)	50	8	28
*L. pneumophila*	30	8	4
*L. pneumophila* (V1 co-culture)	30	8	38
*L. pneumophila* (S2 co-culture)	30	8	44
*L. pneumophila* (M3 co-culture)	30	8	50
*L. pneumophila*	50	8	3
*L. pneumophila* (V1 co-culture)	50	8	3
*L. pneumophila* (S2 co-culture)	50	8	3
*L. pneumophila* (M3 co-culture)	50	8	3

SOURCE: From Dupuy et al. [51]. *Acanthamoeba* were infected with *L. pneumophila. L. pneumophila* were co-cultured with three strains (V1, S2, M3) of *Acanthamoeba.*

**Table 7:** Chlorine Dioxide Inactivation of *L. pneumophila* and Acanthamoeba Trophozoites.

Organism	Temperature (°C)	pH	CT_99.9%_	Reference
*Acanthamoeba* M3	30	8	0.5	[51]
*Acanthamoeba* M3 (infected)	30	8	0.5	[51]
*Acanthamoeba* S2	30	8	2.1 *	[51]
*Acanthamoeba* S2 (infected)	30	8	5.5 *	[51]
*Acanthamoeba* V1	30	8	0.4 *	[51]
*Acanthamoeba* V1 (infected)	30	8	3.5 *	[51]
*Hartmanella vermiformis*	20	7.6–7.8	300 *	[156]
*Legionella* sp.	ND	ND	0.08	[157]
*L. pneumophila*	30	8	0.4	[51]
*L. pneumophila* (V1 co-culture)	30	8	2.8	[51]
*L. pneumophila* (S2 co-culture)	30	8	0.9 **	[51]
*L. pneumophila* (M3 co-culture)	30	8	2.4	[51]

*Acanthamoeba* were infected with *L. pneumophila*. *L. pneumophila* were co-cultured with three strains (V1, S2, M3) of *Acanthamoeba.* * 1 log reduction (i.e., CT_90%_) ** 2-log reduction (i.e., CT_99%_).

**Table 8:** Monochloramine Inactivation of *L. pneumophila* and Acanthamoeba Trophozoites.

Organism	Temperature (°C)	pH	CT_99.9%_
*Acanthamoeba* M3	30	8	19
*Acanthamoeba* M3 (infected)	30	8	20
*Acanthamoeba* S2	30	8	40 *
*Acanthamoeba* S2 (infected)	30	8	47 *
*Acanthamoeba* V1	30	8	23
*Acanthamoeba* V1 (infected)	30	8	24
L. *pneumophila*	30	8	17
*L. pneumophila* (V1 co-culture)	30	8	23
*L. pneumophila* (S2 co-culture)	30	8	22
*L. pneumophila* (M3 co-culture)	30	8	19

SOURCE: From Dupuy et al. [51]. *Acanthamoeba* were infected with *L. pneumophila*. *L. pneumophila* were co-cultured with three strains (V1, S2, M3) of *Acanthamoeba.* * CT99% data.

**Table 9:** Ozone Inactivation of *L. pneumophila* and Amoebae Trophozoites.

Organism	Temperature (°C)	pH	CT 99% (mg-min/L)	Reference
*Naegleria gruberi* (NEG)	25	7	1.3	[172]
*Naegleria gruberi* (NEG)	25	7	<1.6	[173]
*Naegleria gruberi* (1518/1d)	25	7	1.6	[173]
*Naegleria gruberi* (Echirolles)	25	7	<1.6	[173]
*Naegleria* spp. (MO5; C110; An24)	25	7	<1.6	[173]
*Naegleria fowleri*	25	7	<1.6	[173]
*Acanthamoeba polyphaga* (1501/3a)	25	7	2.5	[173]
*Acanthamoeba polyphaga*	20–22	7.5–8	5	[155]
*Acanthamoeba culbertsoni* (A1)	25	7	<1.6	[173]
*Acanthamoeba royreba* (OR)	25	7	<1.6	[173]
*Acanthamoeba* spp. (MR4)	25	7	1.6	[173]
*Vermamoeba vermiformis*	25	ND	<1.6	[173]
*L. pneumophila*	25	ND	60	[170]
*L. pneumophila*	43	ND	55	[170]
*L. pneumophila* serogroup 1	25–45	7.2	0.5	[171]
*L. pneumophila* serogroup 1	25	8	0.95	[171]
*L. pneumophila* serogroup 1	25	8.9	0.65 *	[171]

ND: Not determined, * CT_99.9_ data.

**Table 10:** Efficacy of UV for Inactivation of *Legionella* and Amoebae.

Organism	Fluency (mJ/cm^2^) for Respective Inactivation				Reference
	1 Log	2 Logs	3 Logs	4 Logs	
*Acanthamoeba* sp.	40				[174]
*A. castellani* CCAP 1534/2 (Trophozoites)	32.1		22.7		[176]
*A. castellani* CCAP 1534/2 (Cysts)	45.4		90.9		[176]
*Acanthamoeba* sp. 155 (Trophozoites)	27.6		65.7		[176]
*Acanthamoeba* sp. 155 (Cysts)	34.2		99.2		[176]
*V. vermiformis* CCAP 1534/7A (Trophozoites)	10.7		26		[176]
*V. vermiformis* CCAP 1534/7A (Cysts)	16.8		53.8		[176]
*V. vermiformis* 195 (Trophozoites)	10.1		24.2		[176]
*V. vermiformis* 195 (Cysts)	31.5		76.2		[176]
*L. pneumophila* sg. 1 ATCC 33152	1.7			5.7	[176]
*L. pneumophila* sg. 1 env ^a^	1.7			5	[176]
*L. pneumophila* sg. 7 ATCC 33823	1.7			5	[176]
*L. pneumophila* sg. 8 env ^a^.	1.8			6.1	[176]
*L. longbeachae* ATCC 33462	1.4			6.3	[176]
*L. pneumophila* sg. 1 env			4		[176]
*L. pneumophila* sg. 1 env with *A. castellani* CCAP 1534/2			6		[176]
*L. pneumophila* sg. 1 env with *Acanthamoeba* sp. 155			8		[176]
*L. pneumophila* (25 °C and 43 °C)			30		[170]

^a^ *L. pneumophila* sg. 1 env and *L. pneumophila* sg. 8 env were environmental isolates.

**Table 11:** UV doses (mJ/cm^2^) for inactivation of *L. pneumophila*.

*L. pneumophila* Strain	Lamp Type	1-log	2-log	3-log	4-log
Philadelphia Type 2	LP	0.92	1.84	2.76	No data
Philadelphia 1 (no light repair)	LP	0.5	1	1.6	No data
Philadelphia 1 (with light repair)	LP	2.3	3.5	4.6	No data
Philadelphia 1 ATCC33152	LP	1.6	3.2	4.8	6.5
Philadelphia 1 ATCC33152	MP	1.9	3.8	5.8	7.7

Sources: [175,179,180]. Notes: LP = Low pressure lamps, which have a single output of UV peaking around a wavelength of 254 nanometers. MP = Medium pressure lamps, which have polychromatic (or broad spectrum) output of UV at multiple wavelengths.

**Table 12:** Calculated disinfection CT_99.9%_ (mg-min/L) for *E. coli* C and *M. avium* strains *.

Disinfectant	Control	*M. avium* Strain				
(Culture Condition)	*E. coli* C	A5	1060	1508	5002	5502
Chlorine (M7H9)	0.088 ± 0.003	106 ± 9	204 ± 36	164 ± 28	126 ± 27	51 ± 10
Chlorine (water)	ND	1552 ± 403	1445 ± 238	596 ± 292	962 ± 431	551 ± 290
Monochloramine	73 ± 28	97 ± 9	458 ± 152	548 ± 62	1710 ± 814	91 ± 34
Chlorine Dioxide	0.015 ± 0.003	ND	8 ± 3	ND	11 ± 2	2 ± 0.1
Ozone	0.002 ± 0.002	ND	0.17 ± 0.14	ND	0.12 ± 0.01	0.10 ± 0.01

* Cells were exposed to the disinfectants in demand-free phosphate buffer (pH 7.0) at 23 °C. ND, not determined. Adapted from Taylor et al. [186].

**Table 13:** Comparison of disinfection conditions (CT_99.9%_ in mg-min/L) for Giardia cysts and *M. avium*.

Disinfectant	*Giardia* Cysts	*M. avium*
Chlorine	46	130
Monochloramine	700 *	580
Chlorine Dioxide	11	7
Ozone	0.48	0.13

Data are for pH 7.0, 23–25 °C. Average *M. avium* data based on Taylor et al. [186]. * Extrapolated.

**Table 14:** Comparison of free chlorine disinfection (CT_99%_ in mg-min/L) for Giardia cysts and *M. fortuitum*.

Temperature (°C)	pH	*Giardia* Cysts	*Mycobacterium* *fortuitum*
5	6.0	70	>320–1000
5	7.0	99	320–630
5	8.0	144	>320–>1000
15	6.0	35	90–320
15	7.0	50	500–>1000
15	8.0	72	>320–1000
25	6.0	17	50–>320
25	7.0	25	130–>320
25	8.0	36	130–>320

Data for free chlorine at 1.0 mg/L, based on Jacangelo et al. [219].

**Table 15:** Free chlorine disinfection of mycobacteria.

Organism	CT Value (mg-min/L)	Log Inactivation
*M. fortuitum*	2	0.04
	42	2.9
*M. chelonae*	2	0.03
	10.5	2.5
	21	4.75
	42	2.8
*M. gordonae*	2	0.07
*M. scrofulaceum*	2	0.08

Data based on Carson et al. [190,214]. Data are for pH 7.0, 22–25 °C.

**Table 16:** Chloramine disinfection of mycobacteria for 0.5 log inactivation.

Organism	Chloramine (mg/L)	Contact Time (min)	CT Value (mg-min/L)
*M. avium* 743	1.0	600	600
	3.0	240	720
	6.5	30	195
*M. avium* 723	1.0	600	600
	3.0	240	720
	6.5	30	195
*M. intracellulare*	1.2	258	310
	3.0	120	360
	6.5	30	195
*M. chelonae*	1.6	48	76
	3.0	36	108
	6.5	6	39
*M. gordonae*	1.6	60	96
	3.0	40	120
	6.5	10	78
*M. kansasii*	1.2	60	72
	3.0	10	30
	6.5	9	58
*M. fortuitum*	1.5	36	54
	3.0	24	72
	6.5	2	19.5

Data based on Pelletier et al. [221]. Data are for pH 7.0, 17 °C.

**Table 17:** Inactivation of mycobacteria by ultraviolet light.

Strain	Log Inactivation	UV Dose (mJ/cm^2^)	Reference
*M. tuberculosis* H37Rv	1	5.7	[222]
*M. tuberculosis* Erdman	1	2.4	[224]
*M. tuberculosis*	1	2.8	[225]
*M. avium* DM9 *	2	7	[223]
*M. avium-intracellulare* T-931-72 *	2	14	[225]
*M. avium-intracellulare* T-931-72 *	1	8.4	[225]
*M. avium subsp. hominissuis, MC02*	1	6	[226]
*M. avium subsp. hominissuis, MC02*	4	20	[226]
*M. bovis* BCG	1	2.4	[224]
*M. fortuitum* strain 1	1	3.2	[222]
*M. fortuitum* strain 2	1	8.9	[222]
*M. fortuitum* strain 56	1	6.8	[225]
*M. phlei* strain 44	1	7.6	[225]
*M. kansasii* (avg. of 6 strains) *	1	13.3	[225]
*M. marinum* strain 1	1	17.8	[222]
*M. marinum* strain 1	1	17.0	[222]
*M. flavescens*	1	12.0	[225]
*M. smegmatis*	1	24.3	[222]
*M. smegmatis*	1	10.8	[225]
*M. fortuitum*	3	50	[227]

* Data following photoreactivation.

**Table 18:** M. avium and HPC biofilm levels in a pilot distribution system supplied with various levels of AOC.

Weeks Sampled	Baseline, 42 μg/L		85 μg/L AOC		103 μg/L AOC		213 μg/L AOC	
	HPC	*M. avium*	HPC	*M. avium*	HPC	*M. avium*	HPC	*M. avium*
1	5.27	4.24	5.04	3.86	5.62	4.41	5.08	3.68
4	5.95	4.10	6.27	3.89	6.40	4.03	6.82	4.89
9	5.99	3.17	6.08	4.44	6.64	3.85	7.16	5.36
10	5.51	4.90	6.31	4.44	6.74	4.96	6.88	5.96
11	6.51	5.07	6.33	4.27	6.50	3.91	6.98	4.04
Avg	5.85	4.30	6.01	4.18	6.38	4.23	6.58	4.79

Values are log cfu/cm^2^, AOC values are geometric means, no disinfectant was applied. pH 7.2, 24 °C. Adapted from Norton et al. [104].

**Table 19:** Efficacy of various disinfectants for *P. aeruginosa*. Adapted from Bedard et al. 2026.

Disinfectant	Suspended or Biofilm Cells	Experimental Scale	Disinfectant Dose	Contact Time (min)	Initial Cell Concentration (cfu/mL)	Log Reduction	Strain, Resistance or Recovery
Chlorine	Suspended	Laboratory	0.5 mg/L Cl_2_/L	<1	10^6^	4	PAO1
		Laboratory	0.5 mg/L Cl_2_/L	30	8 × 10^−1^	0.6	Env–river water
		Laboratory	0.1–0.6 mg/L Cl_2_/L	5	10^6^	0.4–4.3	Env–water system biofilm
	Biofilm	Laboratory	0.5 mg/L Cl_2_/L	30	10^6^	1.7	PAO1–biofilm
		Laboratory	5.8 mg/L Cl_2_/L	60	nd	2	Env
Monochloramine	Suspended	Laboratory	2 mg/L Cl_2_/L	30	10^6^	5	PAO1
	Biofilm	Laboratory	4 mg/L Cl_2_/L	60	3.8 × 10^12^ cfu/m^2^	4	ERC1–hydraulic system biofilm
Chlorine Dioxide	Suspended	Laboratory	0.5 mg/L Cl_2_/L	30	10^7^	5	Env
		Laboratory	1.5 mg/L Cl_2_/L	30	10^7^	7	Env
	Biofilm	Laboratory	1.5 mg/L Cl_2_/L	30	10^7^	<1	Env
Silver ions	Suspended	Laboratory	5 mg/L	20	3 × 10^7^	2	PAO1 wild-type BAA-47; resistance over time
		Laboratory	0.08 mg/L	720	3 × 10^6^	6	PAO1–biofilm
		Laboratory	0.1 mg/L	480	10^6^	5.5	Env
	Biofilm	Laboratory	5 mg/L	20	6.3 × 10^7^	1	PAO1 wild-type BAA-47; resistance over time
		Laboratory	10 mg/L	30	10^6^	0.06	PAO1–biofilm
Copper ions	Suspended	Laboratory	0.6 mg/L	600	10^6^	6	Env–plumbing biofilm, full recovery
		Laboratory	0.1 mg/L	90	3 × 10^6^	6	Env
		Laboratory	2 mg/L	300	10^6^	6	PAO1 wild type
	Biofilm	Laboratory	16 mg/L	300	3 × 10^7^	3.5	PAO1 wild-type; resistance to copper observed
Ozone	Suspended	Laboratory	0.6 ppm	6	10^6^	1	Env
			3.14 ppm	2	10^6^	4	Env
		Laboratory	0.37 ppm	0.5; 5	OD_600_ = 1.75–2.0	1.07;1.4	ATCC27853
Thermal shock	Suspended	Hospital	70 °C	30	not applicable		Env strains–contamination at the tap eliminated after thermal treatment
		Hospital	75 °C	60	not applicable		
	Biofilm	Laboratory	65 °C	2	10^8^ cfu/m^2^	5	PAO1
		Laboratory	85 °C	1	4 × 10^4^ cfu/m^2^	2–3	ATCC9027

Env = environmental isolate. See Bedard et al. [252] for full details.

**Table 20:** Summary of Opportunistic Pathogens in Drinking Water Distribution Systems.

Opportunistic Pathogen	Risk from Tap Water	Availability of Treatment	Guidelines for Control
*L. pneumophila*	+++	+++	+++
*M. avium*	++	+	+
*P. aeruginosa*	+	+++	++
*A. hydrophila*	+	+++	+
*K. pneumoniae*	+	+++	+
*S. marcescens*	+	+++	+
*B. pseudomallei*	+	+++	+
*A. baumannii*	+	+++	+
*S. maltophilia*	+	+++	+
*A. butzleri*	+	+++	+
*N. fowleri*	++	+++	++
*Acanthamoeba*	+	+++	+

+ little information available, ++ moderate information available, +++ extensive information available.

## Data Availability

Not applicable.
